# Biradical *o*-iminobenzosemiquinonato(1−) complexes of nickel(ii): catalytic activity in three-component coupling of aldehydes, amines and alkynes†

**DOI:** 10.1039/d0ra10248b

**Published:** 2021-04-06

**Authors:** Mina Nasibipour, Elham Safaei, Ali Moaddeli, Marziyeh Sadat Masoumpour, Andrzej Wojtczak

**Affiliations:** Department of Chemistry, College of Sciences, Shiraz University 71454 Shiraz Iran; Department of Chemistry, Estahban Higher Education Center Estahban 74519-44655 Iran; Nicolaus Copernicus University, Faculty of Chemistry 87-100 Torun Poland

## Abstract

The six-coordinated bis-*o*-iminosemiquinone complex, NiL_2_^BIS^, in which L^BIS^ is the *o*-iminosemiquinone 1-electron oxidized form of the tridentate *o*-aminophenol benzoxazole-based ligand H_2_L^BAP^, was synthesized and characterized. The crystal structure of the complex reveals octahedral geometry with a NiN_4_O_2_ coordination sphere in which Ni(ii) has been surrounded by two tridentate L^BIS^ ligands. This compound exhibits (*S*_Ni_ = 1) with both spin and orbital contribution to the magnetic moment and antiferromagnetic coupling between two electrons on two L^BIS^ ligands which results in a triplet spin ground state (*S* = 1). The electronic transitions and the electrochemical behavior of this open-shell molecule are presented here, based on experimental observations and theoretical calculations. The electrochemical behavior of NiL_2_^BIS^ was investigated by cyclic voltammetry and indicates ligand-centered redox processes. Three-component coupling of aldehydes, amines and alkynes (A^3^-coupling) was studied in the presence of the NiL_2_^BIS^ complex, and the previously reported four-coordinated bis-*o*-iminosemiquinone NiL_2_^NIS^. Furthermore, among these two *o*-iminobenzosemiquinonato(1−) complexes of Ni(ii) (NiL_2_^NIS^ and NiL_2_^BIS^), NiL_2_^NIS^ was found to be an efficient catalyst in A^3^-coupling at 85 °C under solvent-free conditions and can be recovered and reused for several cycles with a small decrease in activity.

## Abstract 2

The six-coordinated bis-*o*-iminosemiquinone complex, NiL_2_^BIS^, in which L^BIS^ is the *o*-iminosemiquinone 1-electron oxidized form of the tridentate *o*-aminophenol benzoxazole-based ligand H_2_L^BAP^, was synthesized and characterized.

## Graphical abstract

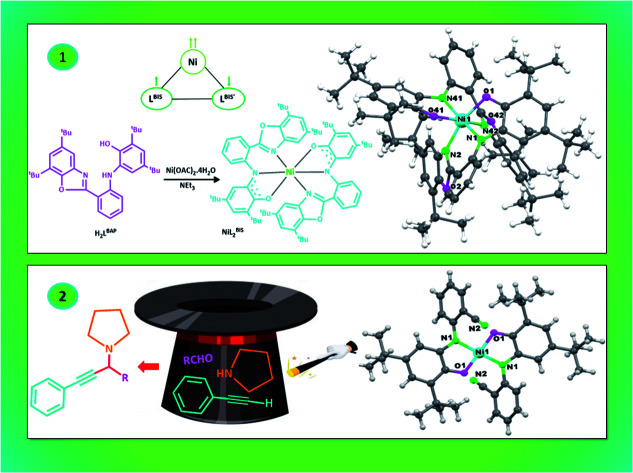

## Introduction

Ligands have the ability of reverse accepting and donating electrons and, therefore, modified redox transformations can be assumed as “electron reservoir” ligands. This property allows electron reservoir ligands to store redox equivalents and consequently, has generated important interest in their multi-electron reactivity features.^2^

One of the classes of ligands which has electron reservoir ability, is redox-active ligands with different oxidation states. When some of these ligands are coordinated to some metal centers and produce complexes, both the metal and the ligand lack defined oxidation states. It means that the mentioned ligands bound to the metal ion could have a different oxidation state and significantly influence the oxidation state of the metal center. These ligands are known as (redox) non-innocent ligands. The interesting part in complexes of non-innocent ligands is that, the frontier orbitals of transition-metal and ligand are close in energy which leads to powerful mixing between these orbitals and it is difficult to assign the oxidation states to metal and ligand components alone.

The existence of redox-active ligands with different oxidation states and the cooperation of these ligands with the metal ion center causes the tuning of oxidation states of the central metal which is the key requirement to reach both developed catalytic activity and improved applicability of the overall complex in catalytic and enzymatic reactions.^3^

**Scheme 1:**
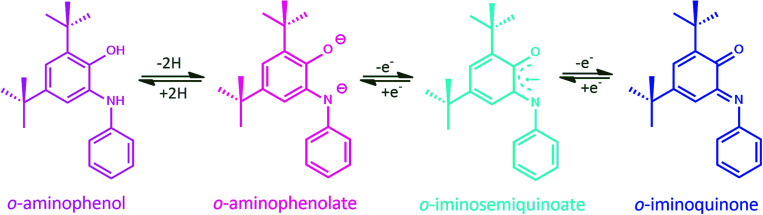
Oxidation states of the bidentate *o*-aminophenol.

Among the numerous kinds of non-innocent ligands, *o*-amidophenolates due to their archetypal coordination abilities and spectroelectrochemical properties, have attracted significant attention. This non-innocent ligand can exist in the completely reduced closed-shell aromatic mono- or dianions of *o*-aminophenolate, or organic, open-shell radical (*S*_rad_ = 1/2) of one-electron oxidized *o*-iminobenzosemiquinone or the closed-shell neutral fully oxidized *o*-iminobenzoquinone (Scheme 1).^4^

Therefore, designing and synthesis of the *o*-aminophenol ligand complexes as unique examples of non-innocent ligands, have been studied considerably and up to now, a number of examples of transition metal complexes (Cu, Pd, Ni, Ir, Ru, Os, Mo, V, Fe, Co, Mn) with two *o*-iminobenzoquinone ligands have been synthesized and characterized due to their structure properties and magnetism.^5^

**Scheme 2:**
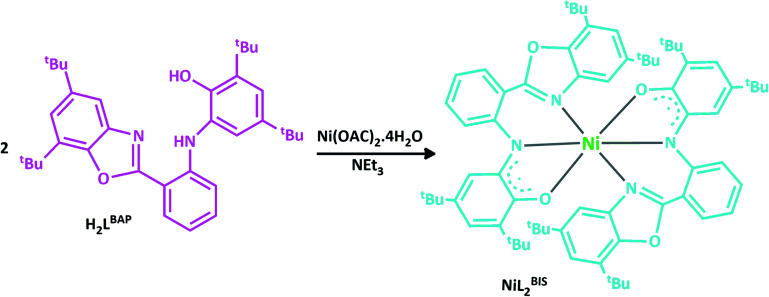
The structure of H_2_L^BAP^ and the associated Ni^II^L_2_^BIS^ complex.

We present here the synthesis and characterization of nickel(ii) complex, NiL_2_^BIS^, which combines two *o*-aminophenol benzoxazole-based ligands that acquire a non-innocent character (Scheme 2). Ni^II^ central atom in this complex and in some similar reported complexes of CuL^BIS^X, X = (Cl, OAC),^6*a*^ X=(Br^−^, I^−^, N_3_^−^, NO_3_^−^)^6*b*^ and Cu(NNO^ISQ^)^6*c*^ is supported by deprotonated (*o*-iminosemiquinone) form of H_2_L^BAP^.

Reaction of Ni(OAC)_2_.4H_2_O with *o*-aminophenol H_2_L^BAP^ in acetonitrile and in the presence of NEt_3_ leads to the formation of the desired complex.

**Scheme 3:**
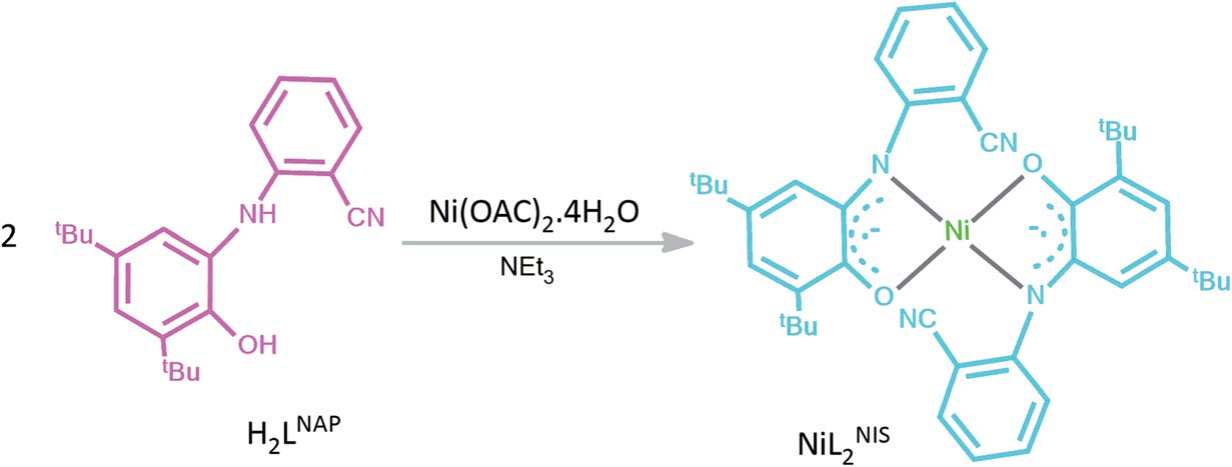
The structure of H_2_L^NAP^ and the associated Ni^II^L_2_^NIS^ complex.^1^

**Scheme 4:**
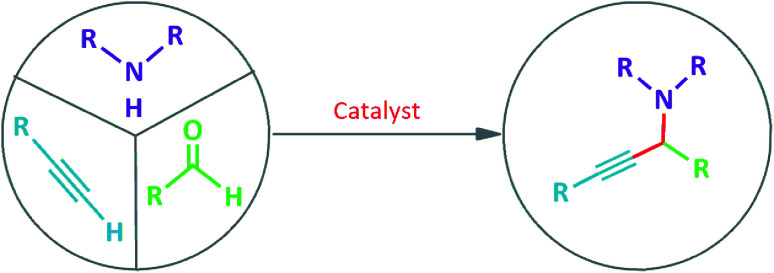
A^3^-coupling or the aldehyde–alkyne–amine reaction which produces propargyl-amine.

As a part of our ongoing effort we try to investigate the catalytic activity of NiL_2_^BIS^ complex and the previously reported four-coordinated bis-*o*-iminosemiquinone of NiL_2_^NIS^^1^ (in which L^NIS^ is the bidentate *o*-iminosemiquinone 1-electron oxidized by 2-amino-4,6-di-*tert*-butyl-phenol ligand of L^NAP^), (Scheme 3), and the comparison between these two Ni(ii) iminosemiquinone complexes in synthesis of propargylamines from three-component coupling of aldehydes, amines and alkynes, A^3^-coupling reactions (Scheme 4).

Propargylamines are useful building blocks for the synthesis of numerous nitrogen-containing heterocyclic compounds, and also important intermediates for preparation of natural complex products and active bio-molecules.^7^ Typically, propargylamines are synthesized by the nucleophilic addition of a metal alkynylide to C=N electrophiles which often needs stoichiometric value of extremely active organometallic reagents like Grignard reagents,^8^ organolithium^9^ and organozinc reagents.^10^ Therefore, it is less attractive in terms of low endurance of functional groups, operational complexity and harsh reaction conditions.

For the last decade, transition metal catalyzed coupling of aldehyde, alkyne, and amine that is usually referred as A^3^-coupling, has received greater attention due to its atom economy, step efficiency, and high chemical selectivity.^11^ This reaction was recommended to be carried out *via* the addition of *in situ* generated metal-alkynylide to iminium ion, that is formed *in situ*, *via* a reaction between amine and aldehyde and water molecule is the only side product.

Transition metal complexes, particularly coinage metal complexes (Ag, Cu and Au), and also In, Zn, Ni, Fe, Ir, Co, Mn, Bi, Hg and Cd have been established as the catalysts for this reaction, among which, there is an increasing interest for Ni catalysts due to their abundance and low costs. In this regard we decided to study the catalytic activity of two Ni(ii) complexes for the mentioned reaction and compare their efficiency in A^3^-coupling based on our observations.

## Results and discussion

### Synthesis and general characterization of NiL_2_^BIS^

The *o*-aminophenol H_2_L^BAP^, was synthesized and purified according to the literature by the reaction of 2-amino benzyl amine and 3,5-di-*tert*-butyl-quinone (DTBQ) in 2 : 1 molar ratio.^6*a*^ The complex NiL_2_^BIS^ was synthesized in good yield, by stirring Ni^II^(OAc)_2_·4H_2_O, H_2_L^BAP^ and Et_3_N in CH_3_CN at room temperature. Slow evaporation from 1 : 2 MeOH/CH_2_Cl_2_ afforded NiL_2_^BIS^ narrow cubic crystals.

The identification of the complex was confirmed by elemental analysis, IR and single-crystal X-ray structural analysis, temperature-dependent magnetic studies and cyclic voltammetry studies.

In the IR spectrum of the NiL_2_^BIS^ complex, the sharp and strong *ν*_O–H_ and *ν*_N–H_ stretch band of the ligand disappears, which confirms the L^BIS^ ligation to the Ni(ii) center. All the vibrations of the ligand, *ν̂* = 1164 cm^−1^ (C–N stretching), *ν̂* = 1614 cm^−1^ (C=C stretching) *ν̂* = 1470 (C=N stretching) and the *tert*-butyl groups bands at *ν̂* = 2962 cm^−1^ exist in the IR spectrum of the complex, that confirms the presence of the ligand in the structure of complex (Fig. S1†).

### Crystal structure of NiL_2_^BIS^ complex

**Fig. 1:**
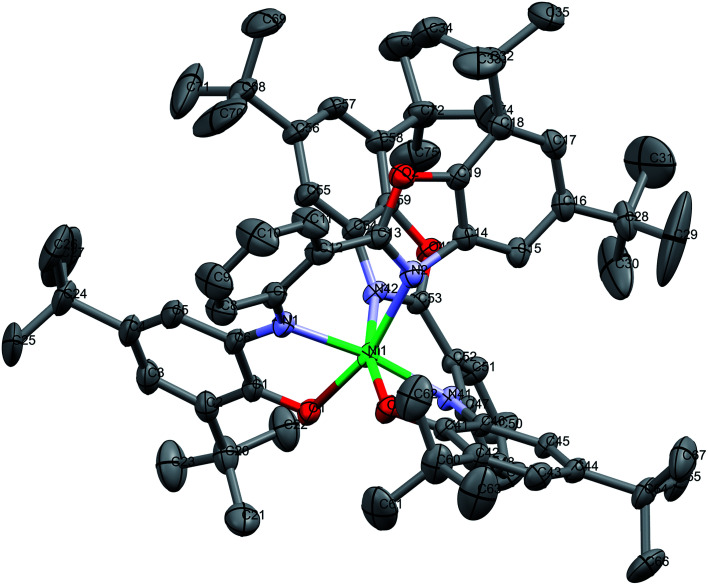
Molecular structure of Ni^II^L_2_^BIS^, H atoms have been omitted for clarity. Thermal ellipsoids are set at 30% probability.

**Fig. 2:**
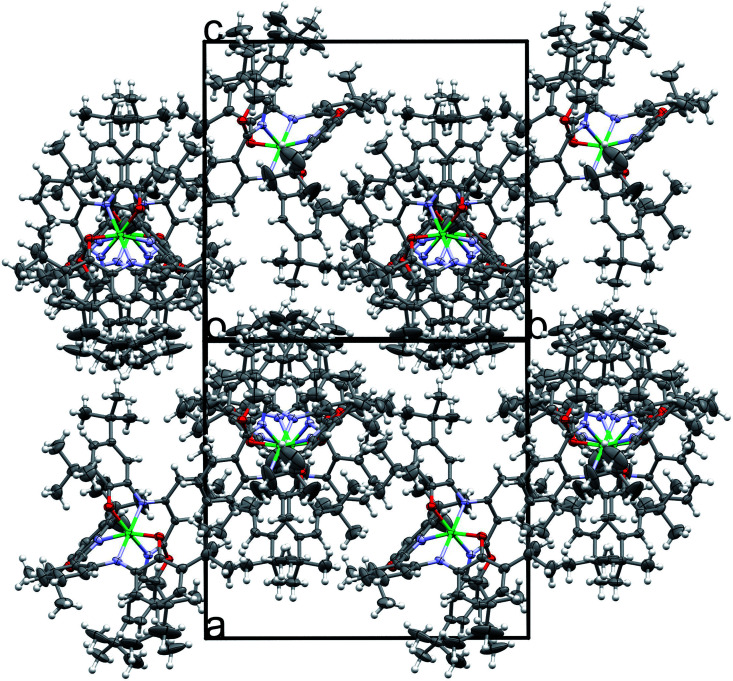
Projection of the unit cell of Ni^II^L_2_^BIS^ revealing the solvent accessible voids (SAV).

In Matte crimson crystals of NiL_2_^BIS^ complex, the asymmetric unit of the reported structure consists of the single molecule of Ni^II^L_2_^BIS^ (Fig. 1). The voids are found in the crystal lattice (solvent accessible voids, 18.4% of the unit cell) with no interpretable electron density (Fig. 2) (see Methods). The diffraction experiments and the structure refinement are summarized in Table 1. The selected bond lengths and angles are given in Table 2. The complete crystallographic data, bond lengths and angles and torsion angles are given in Table S1, S2 and S3 respectively.†

**Table 1:** Crystallographic data for Ni^II^L_2_^BIS^

Empirical formula	C_70_ H_88_ N_4_ Ni O_4_
Formula weight	1108.15
Crystal system	Monoclinic
Space group	*P*2_1_/*n*
Unit cell dimensions	*a* = 18.1423(6), *b* = 17.5195(5), *c* = 23.9371(9)
	*α* = 90, *β* = 106.001(3), *γ* = 90
Volume	7313.5(4) Å^3^
*Z*	4
Temperature	293(2) K
Density (calculated)	1.006 Mg/m^3^
Crystal size	0.793 × 0.564 × 0.517 mm^3^
Absorption coefficient	0.308 mm^−1^
Reflections collected	52 213
Independent reflections	16 548 [*R*(int) = 0.0442]
Goodness-of-fit on *F*^2^	1.040
Final *R* indices [*I* > 2 sigma(*I*)]	*R*_1_ = 0.0614, w*R*_2_ = 0.1426
*R* indices (all data)	*R*_1_ = 0.1017, w*R*_2_ = 0.1725

**Table 2:** Selected bond lengths [Å] and angles [°] for Ni^II^L_2_^BIS^^a^

Ni1–N41	2.0193(19)	C2–C3	1.375(4)
Ni1–N1	2.027(2)	C4–C5	1.368(5)
Ni1–O41	2.0425(18)	C44–C45	1.375(4)
Ni1–O1	2.0466(19)	O1–C1	1.286(3)
Ni1–N2	2.143(2)	O41–C41	1.283(3)
Ni1–N42	2.153(2)	C6–N1	1.355(4)
N41–Ni1–N1	171.41(9)	C46–N41	1.363(3)
O1–Ni1–N2	163.62(8)	N1–C7	1.398(4)
N42–Ni1–O41	164.88(8)	N41–C47	1.394(3)
O1–Ni1–N1	80.16(8)	C53–N42–Ni1	120.77(16)
O41–Ni1–O1	102.55(8)	C54–N42–Ni1	131.18(17)

a Symmetry transformations used to generate equivalent atoms: #1 − *x*, −*y*, −*z*.

The complex has two L^BIS^ ligands coordinated to the central Ni(ii) in the tridentate manner. Each ligand forms the coordination bonds *via* the phenolate O1, imine N1 and benzoxazole N2, with O1 and N2 positioned trans in the coordination sphere. Therefore, in the reported complex the octahedral coordination sphere NiN_4_O_2_ is found. Due to the relative rigidity of the L^BIS^ molecules, two ligands form the structures approximately perpendicular to each other. The shortest coordination bonds are formed by imine N1 and N41 atoms of both ligands (Table 2). The phenolate O1 and O41 form slightly longer Ni–O bonds, the distances being 2.0466(19) and 2.0426(18) Å, respectively. The Ni bonds to benzoxazole N2 and N42 are longer by 0.14 Å from the ones formed by amine N1/N41. The angle between bonds formed by imine atoms N41–Ni1–N1 is 171.41(9)°. The other trans angles O1–Ni1–N2 163.62(8) and O41–Ni1–N42 164.88(8)° differ significantly from the expected 180°. The angles between the coordination bonds formed by atoms in *cis* positions range from N1–Ni1–O1 80.16(8)° to O41–Ni1–O1 102.55(8)° (Table 2). These values indicate significant deformation of the coordination octahedron, that can be attributed to the rigidity of the tridentate L^BIS^ ligand.

The valence geometry of both L^BIS^ ligands is almost identical. In both phenolic rings, bond lengths C2–C3 and C4–C5, and their equivalents range from C4–C5 1.368(5) to C44–C45 1.375(4) Å, indicating the localization of double bonds in these positions. These bonds are significantly shorter than other C–C bonds in the phenolic rings, which range from 1.427(5) to 1.466(4) Å and reveal significant contributions of single bonds (Table 2). The O1–C1 and O41–C41 bonds lengths of 1.286(3) and 1.283(3) Å, respectively, reveal their double rather than single bond character. The C6–N1 and C46–N41 bonds are 1.355(4) and 1.363(3) Å, respectively, and are shorter by 10*σ* than N1–C7 and N41–C47. Such distribution of the double bonds in the *o*-aminophenole fragment corresponds to *o*-iminosemiquinoate form of both L^BIS^ ligands. That form seems to be consistent with both the bond distribution and the neutral charge of the complex with Ni^II^ center.

The tridentate coordination of L^BIS^ causes the conformational adjustments resulting in the lack of planarity of the ligand. The additional factor affecting the conformation is the presence of bulky *t*Bu substituents at the benzoxazole moieties. Their spatial arrangement in the complex molecule results in the intramolecular interactions of their methyl groups C34 to C69 and C31 to C74 (Fig. 1). The observed twist of both ligands can be quantified with the dihedral angles between best planes of the phenolic and benzoxazole moieties, being 58.82(12) and 46.53(12)° for ligand 1 and 2, respectively. The dihedral angles between the central phenyl ring and phenolic and benzoxazole rings are 51.66(17), 34.89(15)° and 49.01(14), 29.19(13)° for O1–O2 and O41–O42 ligands, respectively.

The intramolecular π⋯π interactions are detected. The benzoxazole five-membered heterocyclic moieties form the gravity centers Cg⋯Cg 3.8891(16) Å interaction. That results in the interactions of two benzoxazole moieties with the distance Cg⋯Cg of 3.8770(14) Å, with the dihedral angle between their planes being 39.21(10)°.

**Fig. 3:**
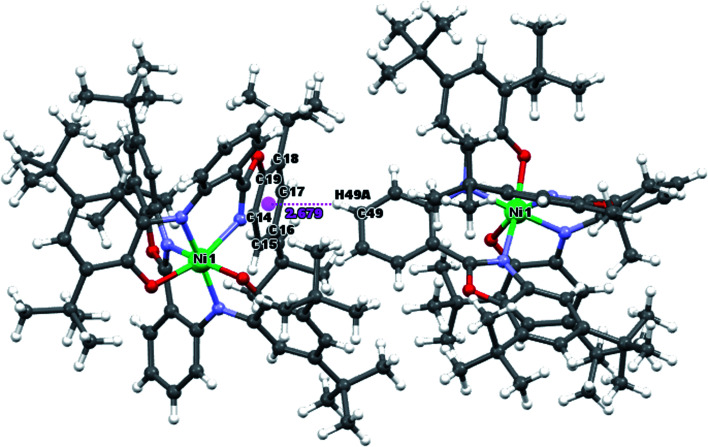
C–H⋯π interaction between Ni^II^L_2_^BIS^ moieties.

The intermolecular C–H⋯π interactions are detected between C49–H49A and C14–C19[3/2 − *X*, 1/2 + *Y*, 1/2 − *Z*] six-membered ring of benzoxazole, with the H⋯Cg distance 2.68 Å and C–H⋯Cg angle 158°. For the interactions of the C49–H49A group with the whole benzoxazole[3/2 − *X*, 1/2 + *Y*, 1/2 − *Z*] moiety, the corresponding values are 2.65 Å and C–H⋯Cg angle 160° (Fig. 3).

The intramolecular hydrogen bonds are shown in Table 3.

**Table 3:** Hydrogen bonds for Ni^II^L_2_^BIS^ [Å and °]^a^

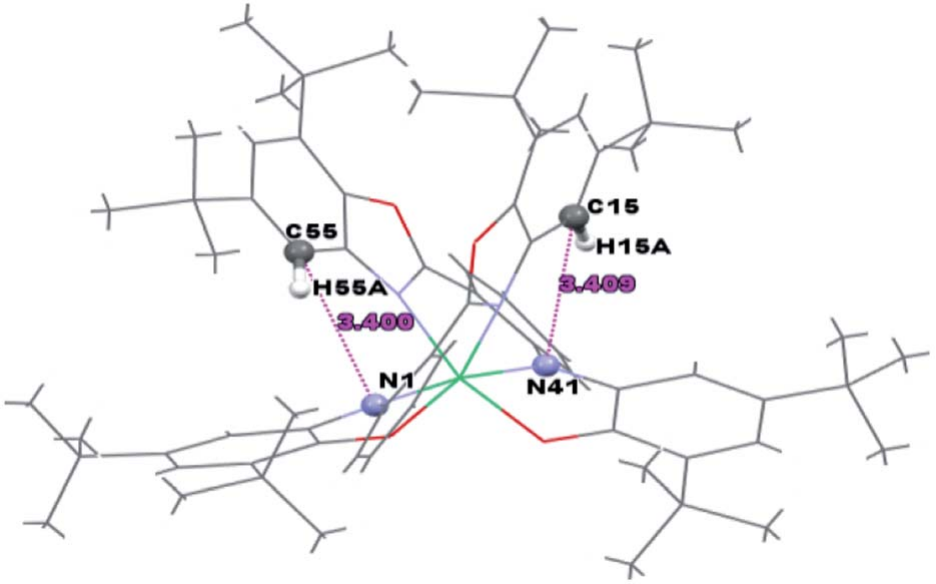	D–H⋯A	d(D–H)	d(H⋯A)	d(D⋯A)	<(DHA)
	C(15)–H(15A)⋯N(41)	0.93	2.70	3.409(4)	133.7
	C(55)–H(55A)⋯N(1)	0.93	2.68	3.400(4)	134.7

a Symmetry transformations used to generate equivalent atoms.

### Magnetic susceptibility measurements

**Fig. 4:**
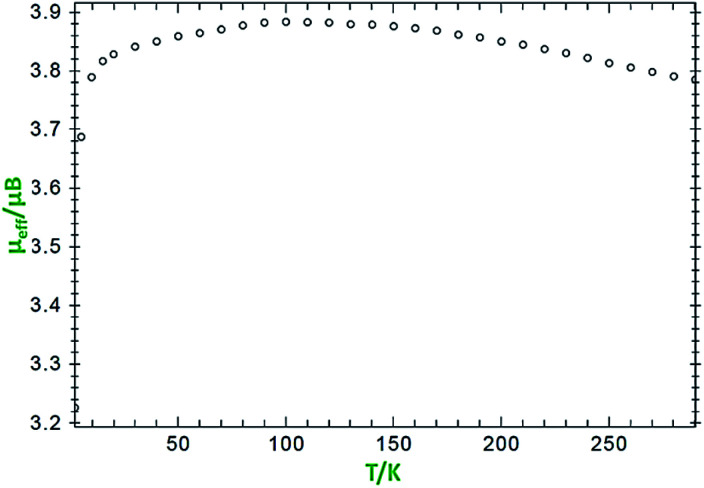
Variation of effective magnetic moment (*μ*_eff_) with variation in temperature for the complex NiL_2_^BIS^.

Variable-temperature magnetic susceptibility measurement for crystalline samples of the ligand H_2_L^BAP^ and NiL_2_^BIS^ complex was carried out with an applied magnetic field of 1000 Oe in the temperature range 1.8–300 K (Fig. 4).

**Scheme 5:**
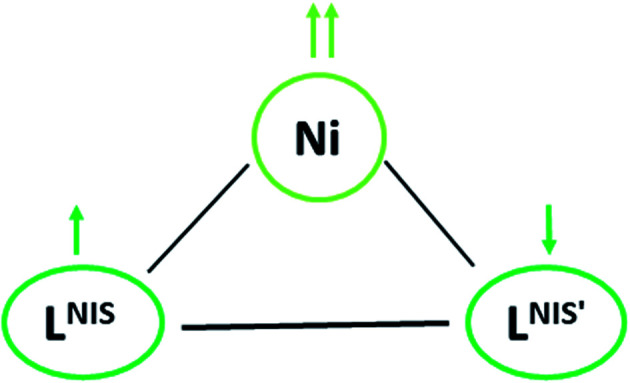
Representation of variation in redox state in NiL_2_^BIS^ complex.

The effective magnetic moment values of NiL_2_^BIS^ change from 3.7 BM (at 1 K) to 3.88 BM (at 125 K) and 3.78 (at 300 K). These values differ from that of the spin-only moment, which amounts to 2.83 BM. This difference between the measured and calculated values results from spin-orbital coupling and displays positive and commonly large deviations from the spin-only contribution of 2.83 BM. The reported compound exhibits (*S*_Ni_ = 1) because of the Ni^II^ center and antiferromagnetic coupling of both tridentate L^BIS^ ligand radicals coordinated to Ni(ii) ion whose spin alignment seems to be [(↑)–(↑↑)–(↓)] (Scheme 5). It indicates that the nickel complex exists in an octahedral triplet ground state. The ground state configuration of Ni(ii) ion in a regular octahedral field is ^3^A_2g_(t^6^_2g_e^2^_g_) and it will be paramagnetic with two unpaired electrons.^12^

### Electrochemistry

The cyclic voltammetry (CV) electrochemical behavior of complex Ni^II^L_2_^BIS^ has been recorded in CH_2_Cl_2_ solution containing 0.10 M NBu_4_ClO_4_ as supporting electrolyte at a glassy carbon as working electrode, and an Ag/AgNO_3_ reference electrode at room temperature.

**Fig. 5:**
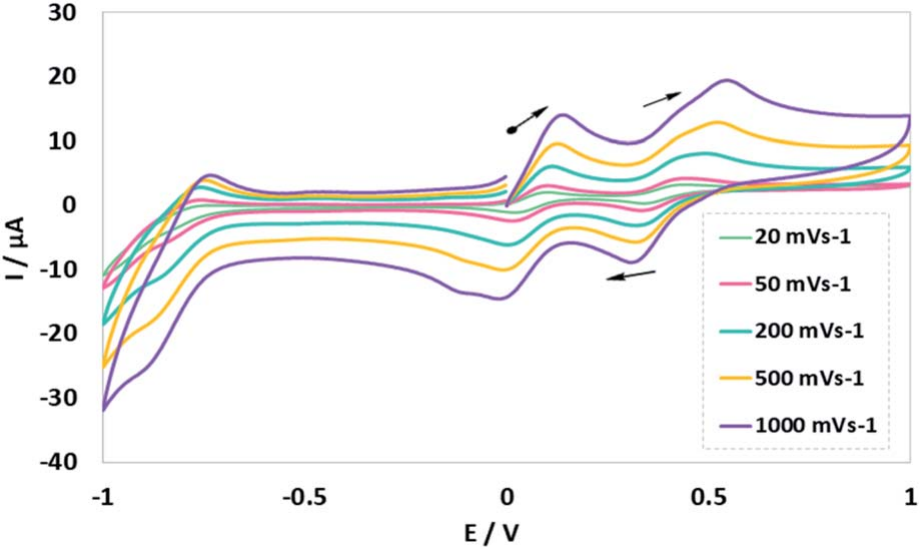
Cyclic voltammograms of NiL_2_^BIS^. Conditions: 1 mM complex, 0.1 M NBu_4_ClO_4_, scan rate 20, 50, 200, 500, 1000 mV s^−1^, CH_2_Cl_2_, 298 K.

**Scheme 6:**
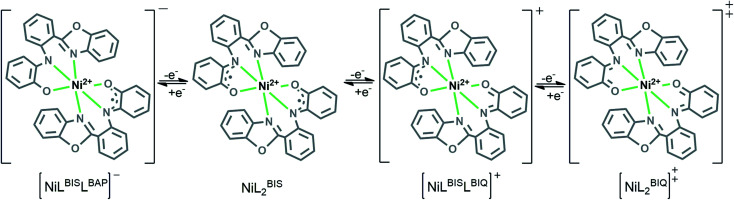
Schematic representation of NiL_2_^BIS^ complex oxidation state variation.

The electrochemical behavior of the complex Ni^II^L_2_^BIS^ is similar to the previously studied *o*-iminobenzoquinone based complexes of Ni(ii),^1^ owing to the presence of three one-electron ligand-centered redox transitions on the voltammogram (Fig. 5 and Table 4). The ligand-centered voltammograms are observed in the positive potential range showing radical-ligand based iminobenzosemiquinone/iminobenzoquinone (Ni^II^L_2_^BIS^/Ni^II^L^BIS^L^BIQ^ and Ni^II^L^BIS^L^BIQ^/Ni^II^L_2_^BIQ^) redox couples and voltammograms observed in the negative potential range corresponding to iminobenzosemiquinone/amidophenoxide redox couples (Ni^II^L_2_^BIS^/Ni^II^L^BIS^L^BAP^) (Scheme 6).

**Table 4:** Redox potentials of NiL_2_^BIS^*versus* Fc^+^/Fc^a^

Compound	*E*_1/2_^1^/*V*	*E*_1/2_^3^/*V*	*E*_1/2_^3^/*V*
NiL_2_^BIS^	−0.7	0.16	0.56

a The potential reported here is the average of anodic and cathodic peak potentials for a reversible process or the peak potential for an irreversible process.

This process corresponds to the following equations (eqn (1)–(3)):1
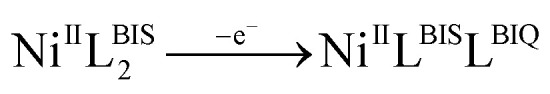
2
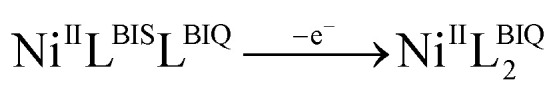
3
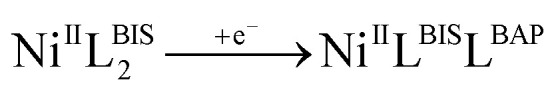


### Electronic spectroscopy

**Fig. 6:**
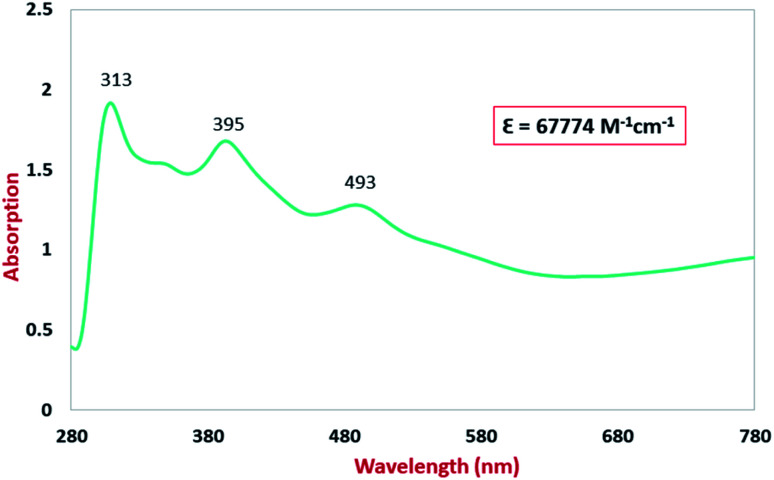
Electronic spectra of 0.02 mM CH_2_Cl_2_ solutions of NiL_2_^BIS^.

The UV-Vis/NIR spectra for the studied compound in CH_2_Cl_2_ solution were recorded in the range of 280–780 nm. The electronic spectrum of the ligand H_2_L^BAP^ is shown in Fig. S2† and the electronic spectrum of the complex Ni^II^L_2_^BIS^ is shown in Fig. 6. Ni^II^L_2_^BIS^ exhibits four absorption bands in the visible, near infrared and near ultraviolet regions (Fig. 6). A ligand π → π* charge transfer was found for the complex in the ultraviolet field of the electronic spectra (313 nm). The electronic absorption band in region 395 nm is consistent with iminosemiquinone (π)-to-Ni–(dπ*), ligand-to-metal charge-transfer (LMCT) transition. The broad electronic absorption band in the region around 493 nm corresponded to metal-to-ligand (MLCT) charge transfer. Also, the extinction coefficient of the ligand and the Ni-complex have been extracted by the plot of the absorption maxima at a selected wavelength *versus* varied concentrations reply to Beer–Lambert law.

We did the fluorescence experiment for the H_2_L^BAP^ ligand and Ni^II^L_2_^BIS^ complex. The ligand was emissive and the complex was non-emissive. The emission spectrum of the ligand is given in Fig. S2.†

## Computational details

### Description of the Ni^II^L_2_^BIS^ structure

Optimized geometries were confirmed to be minima by the frequency analysis. The DFT calculation shows that triplet Ni^II^ octahedral complex, Ni^II^L_2_^BIS^, has the lowest energy. The singlet electronic structure is 33.4 kcal mol^−1^ higher in energy with respect to the triplet solution.

**Fig. 7:**
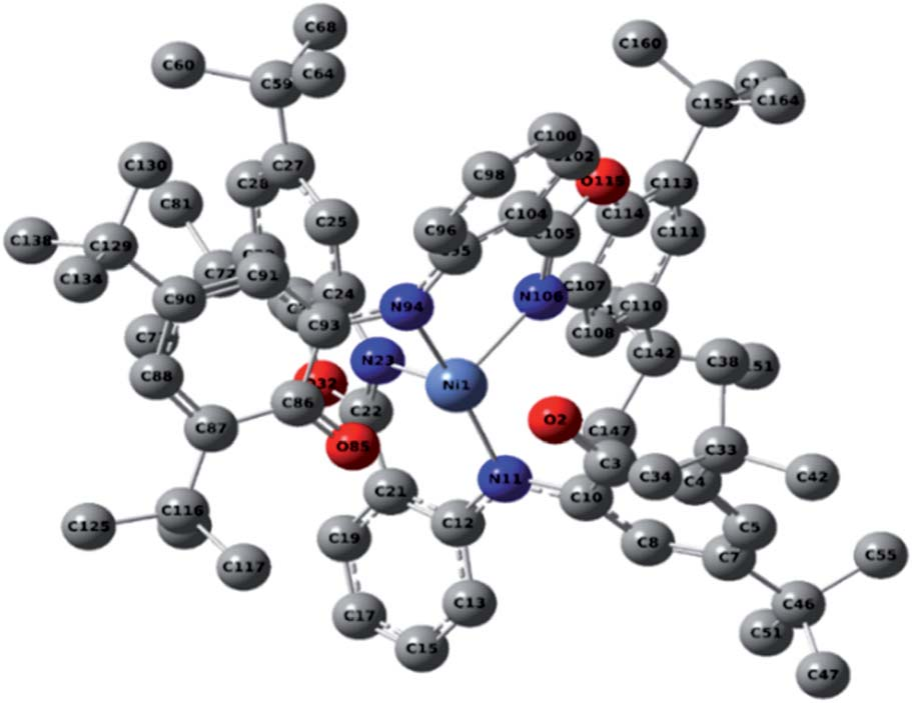
The optimized structure of the Ni^II^L_2_^BIS^ complex. For clarity, hydrogen atoms are omitted.

**Fig. 8:**
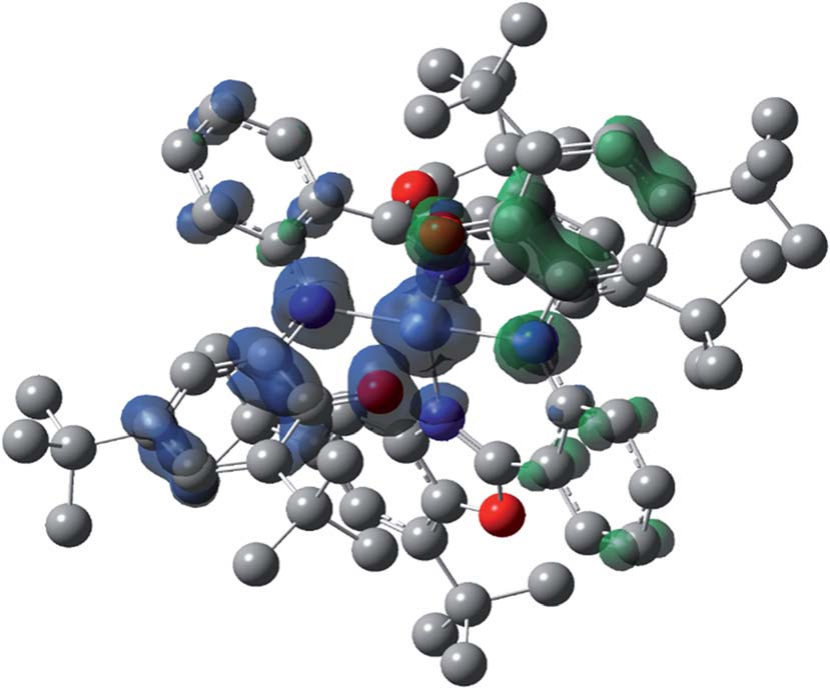
Spin density plot of the Ni^II^L_2_^BIS^ complex.

The optimized structure of Ni^II^ octahedral complex is shown in Fig. 7 and in Table S4.† Some of the selected bond lengths are included. The root-mean-square deviation (RMSD) for the bond lengths from the crystal structure are in the order of 0.02 Å. Therefore, the optimized geometry of the complex is in good agreement with the experimental structure from X-ray crystallography. The C–O, C–N and aryl C–C bond distances of the redox-active fragments of both ligands are similar to the iminosemiquinone (L^BIS^)^1−^ oxidation state reported by Brown.^13^ The predicted spin density for the Ni^II^L_2_^BIS^ complex (Fig. 8) also shows the delocalization of *α* electron density over one ligand and *β* electron density over another ligand in agreement with the iminosemiquinone (L^BIS^)^1−^ oxidation state for both ligands.

### Theoretical analysis of the UV-Vis transitions for Ni^II^L_2_^BIS^

A detailed theoretical study of the UV-Vis electronic absorption spectrum of Ni^II^L_2_^BIS^ complex was carried out using the TD-DFT approach to gain insight into the spectral features of the complex. In order to get an adequate comparison between the theoretical and experimental spectra, we performed self-consistent reaction field (SCRF) calculations in the presence of a solvent using the Polarizable Continuum Model (PCM).^14^ The solvent chosen was that used for the experimental spectrum, that is dichloromethane (CH_2_Cl_2_), with a dielectric constant *ε* = 8.94. The wavelengths, excitation energies, and oscillator strengths of the absorption bands calculated with the TD-DFT methodology are displayed in Table 5. For each transition, molecular orbitals with major contribution are also mentioned in Table 5. The nature of most of the TD-DFT transitions involve the excited states, that are not dominated by one singly excited configuration. For analyzing the nature of such electronic transitions, a Natural Transition Orbital (NTO) analysis^15,16^ has been performed as implemented in Gaussian 16 and the dominant NTO orbital pairs were calculated. The most important advantage of this procedure is that an electronic transition is readily attributed, for the majority of cases, to (at most) two NTO pairs (one occupied and one virtual) that qualitatively depict the electron density reorganization due to the excitation, thus facilitating the assignment of the transition nature. Table 6 gives the NTO pairs describing the transitions for the Ni^II^L_2_^BIS^ complex. The two predicted lower energy bands are assigned as ligand-to-ligand charge transfer (LLCT) transitions (T14 and T34 states). These transitions can be identified as LLCT within the aromatic system of two ligands (π → π* transition). The highest-energy band (T61 state) has mixed character and the electron density migrates form metal-to-ligand (d → π* transition) or ligand to ligand (π → π* transition) groups (mixed MLCT/LLCT state).

**Table 5:** Experimental and calculated absorption properties of the Ni^II^L_2_^BIS^ complex

Exp. *λ*_max_/nm	Tr^a^	Major contribution	Energy/eV (nm)	Oscillator strength	Assignment
493	14	HOMO(*α*) → LUMO+1(*α*) (41%)	2.47 (501)	0.1144	LLCT
		HOMO−3(*β*) → LUMO(*β*) (37%)			
395	34	HOMO−1(*α*) → LUMO+1(*α*) (10%)	3.22 (384)	0.0941	LLCT
		HOMO(*α*) → LUMO+3(*α*) (18%)			
		HOMO−1(*β*) → LUMO+1(*β*) (24%)			
313	61	HOMO−15(*β*) → LUMO(*β*) (28%)	3.92 (316)	0.0376	MLCT/LLCT
		HOMO−14(*β*) → LUMO(*β*) (19%)			
		HOMO−12(*β*) → LUMO(*β*) (12%)			

a Tr = transition number as obtained in the TD-DFT calculation output.

**Table 6:** NTO plots for transitions 14, 33 and 61 of Ni^II^L_2_^BIS^ complex calculated using TD-B3LYP method in dichloromethane solution

Transition	Spin	Donor	Acceptor
T14, *λ* = 501.0 nm, *f* = 0.1144	*α*	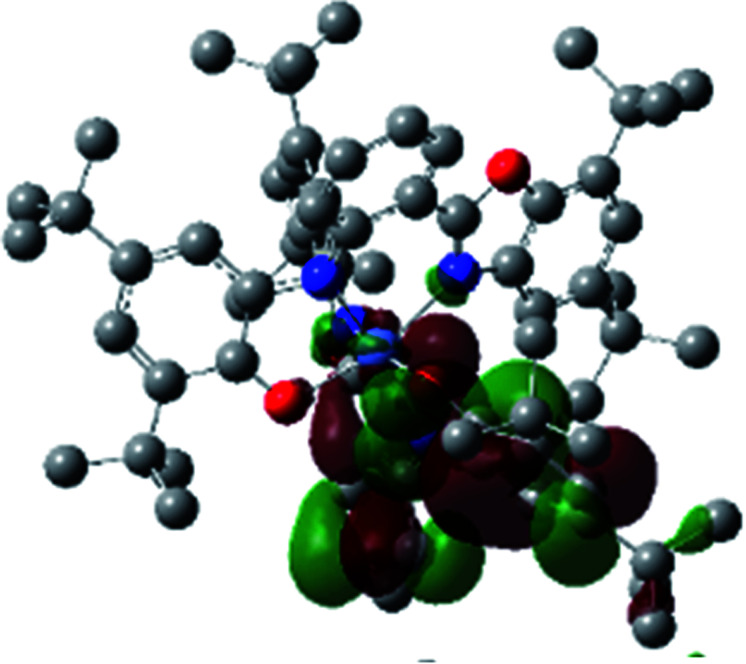	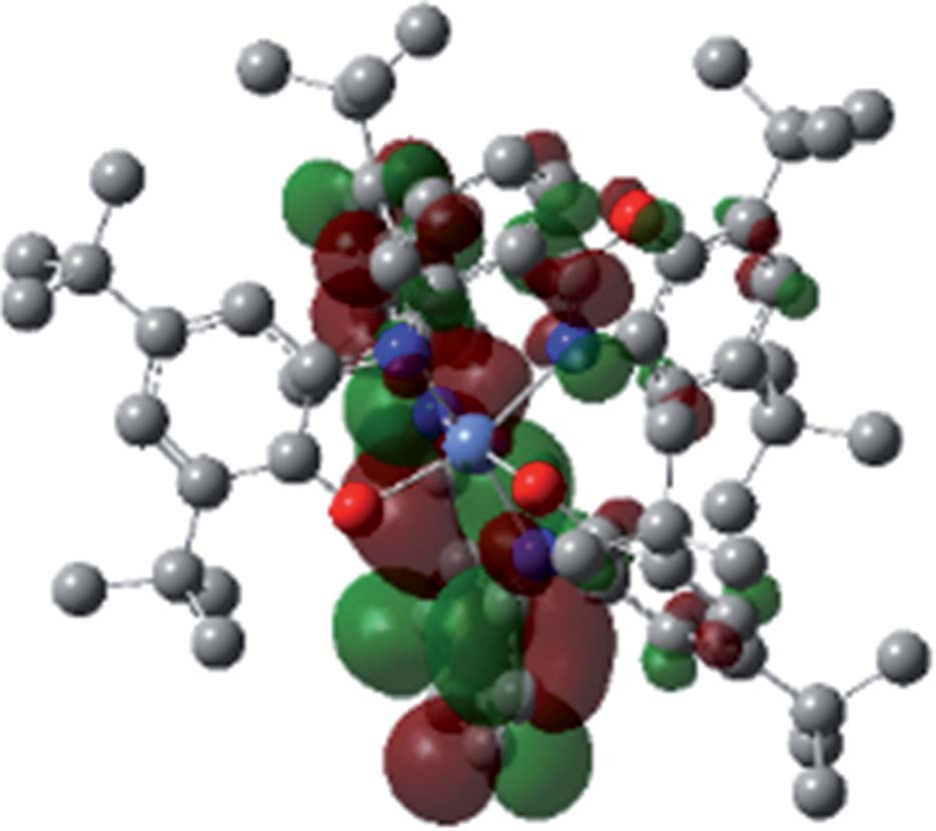
	*β*	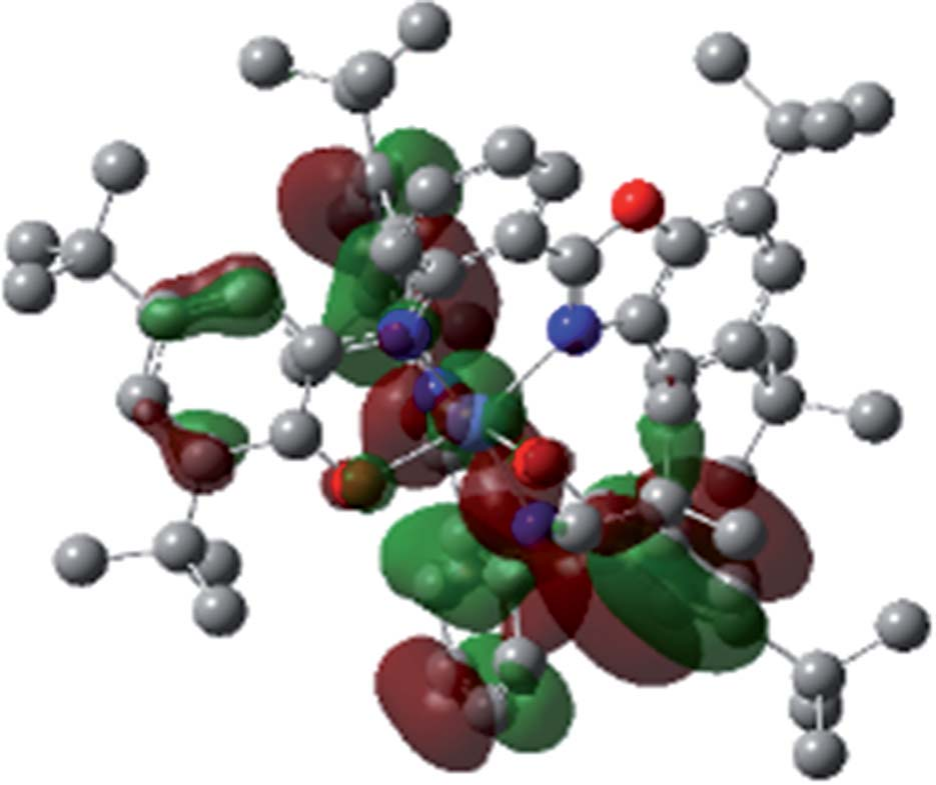	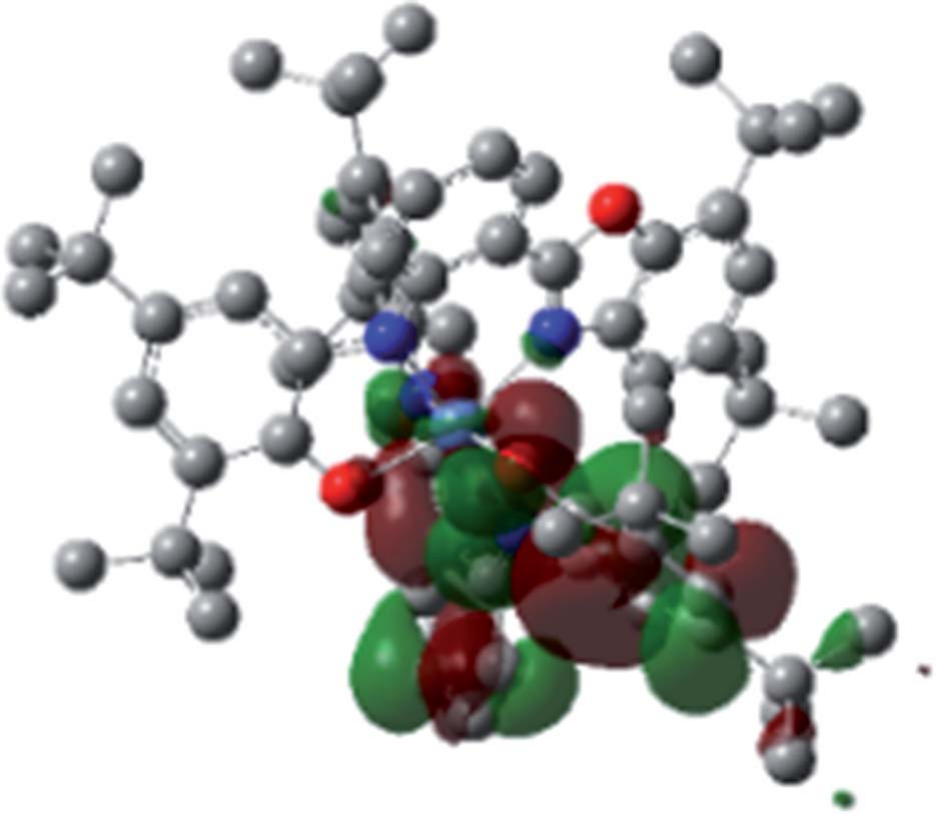
T34, *λ* = 384.1 nm, *f* = 0.0941	*α*	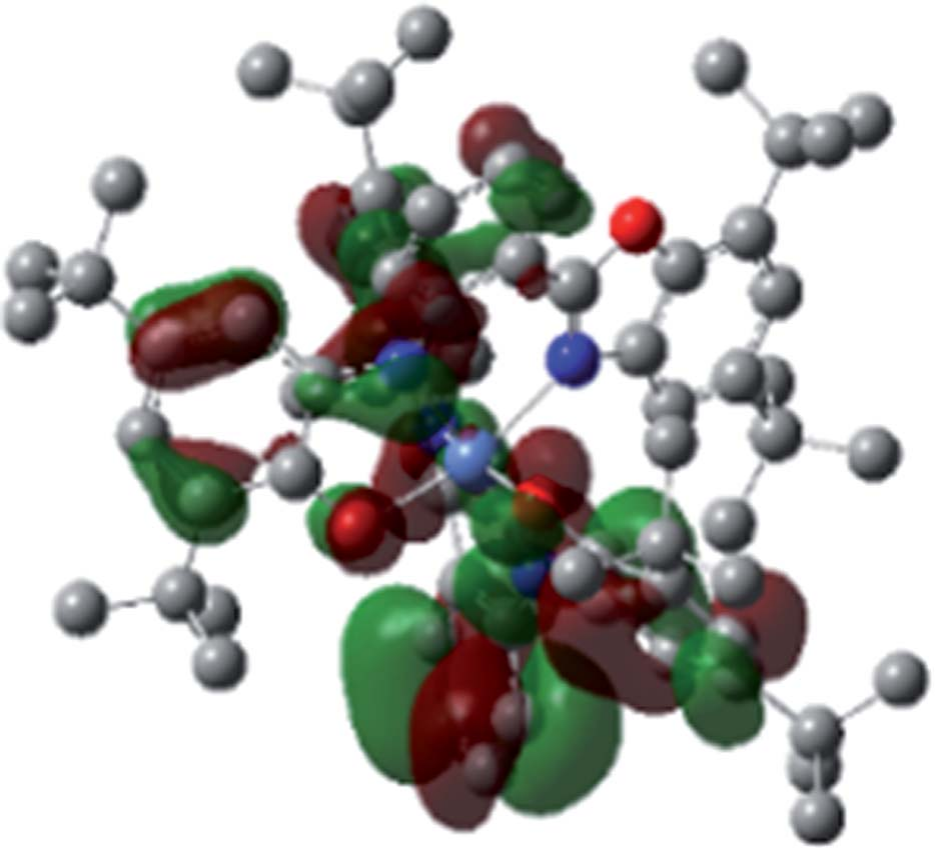	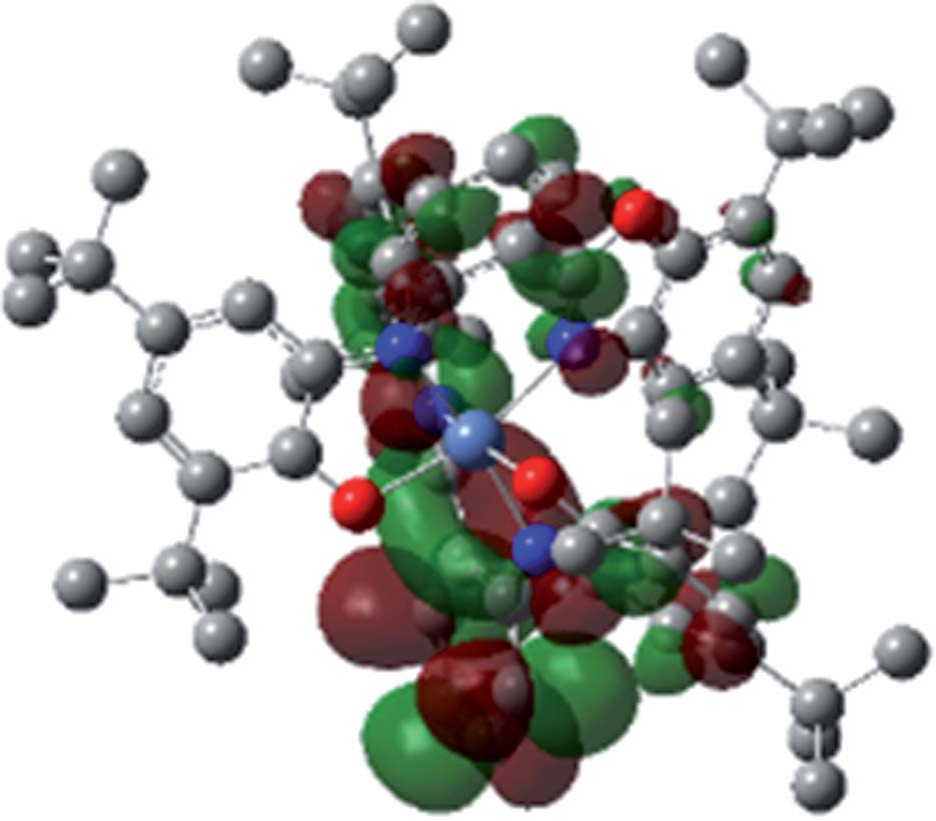
	*β*	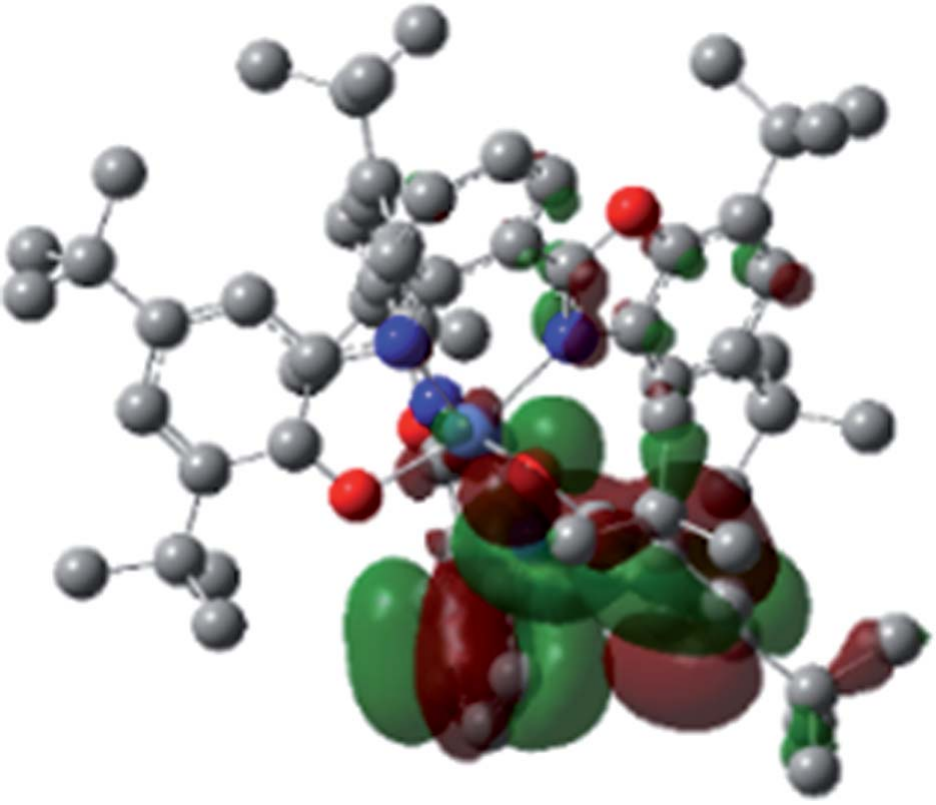	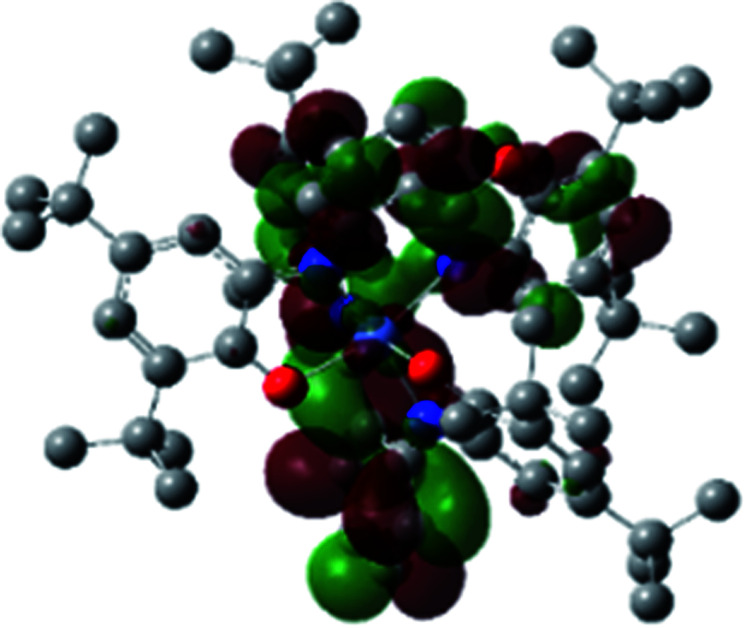
T61, *λ* = 315.9 nm, *f* = 0.0376	*α*	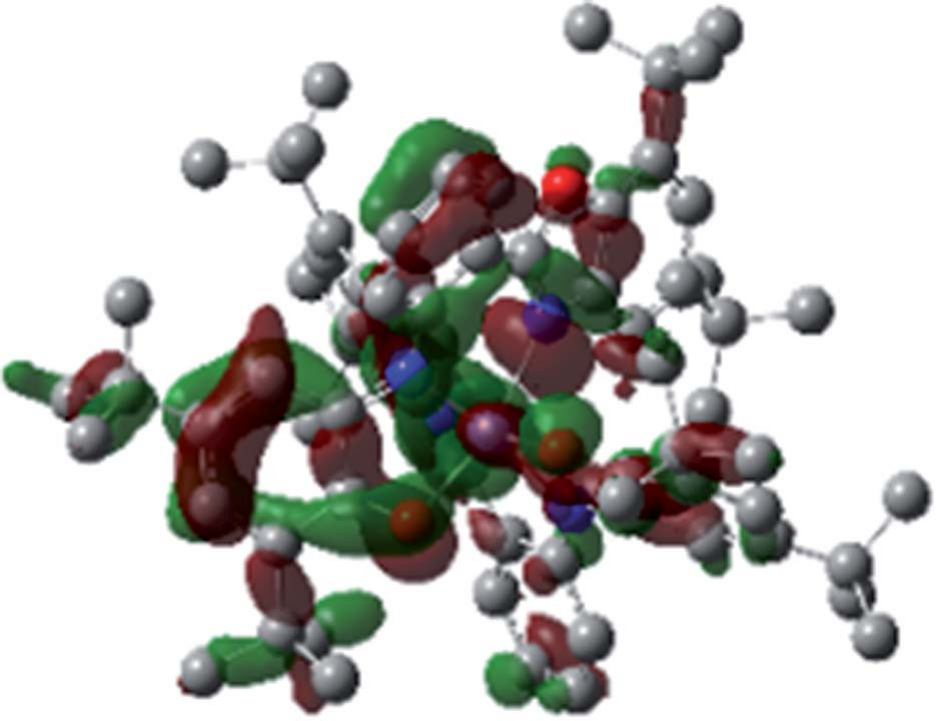	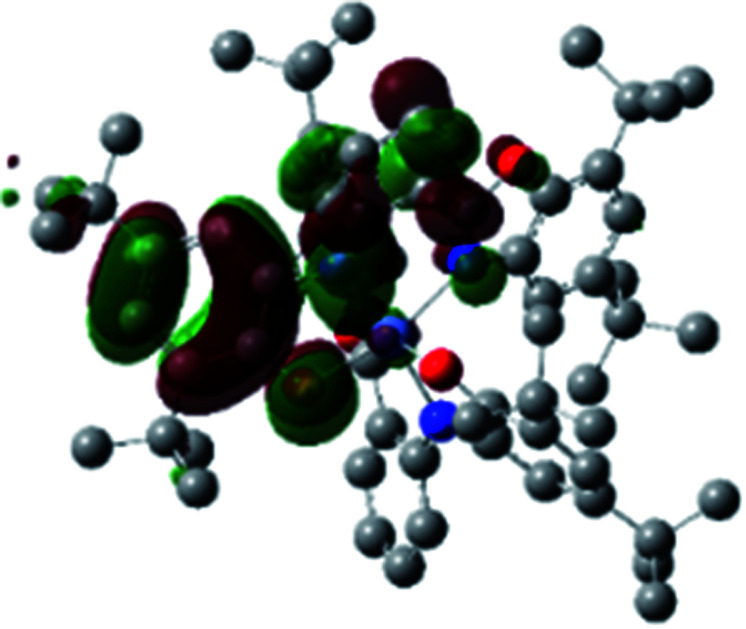
	*β*	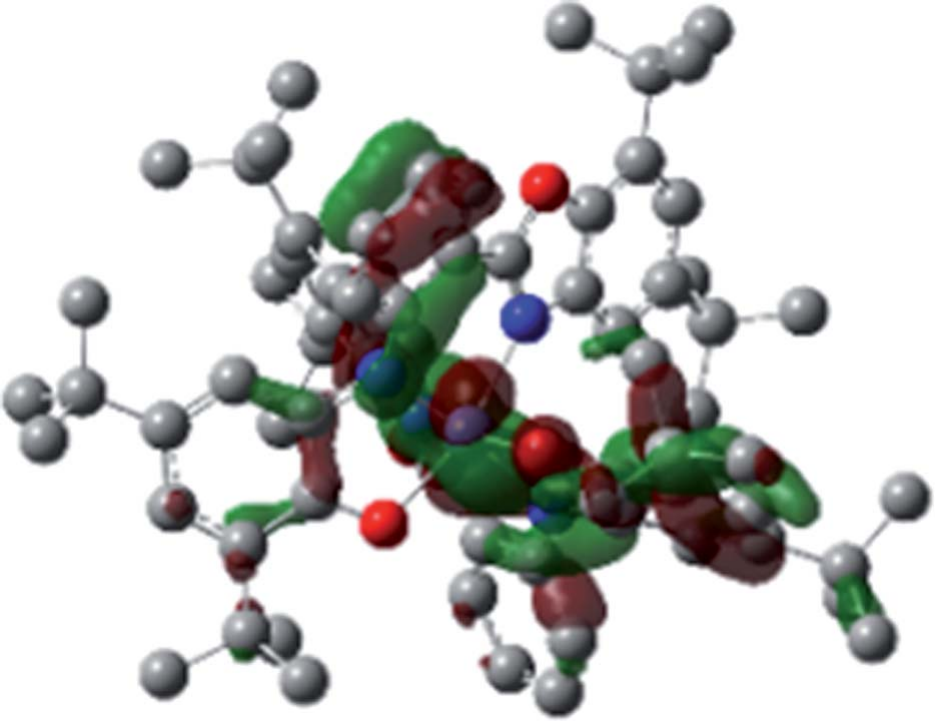	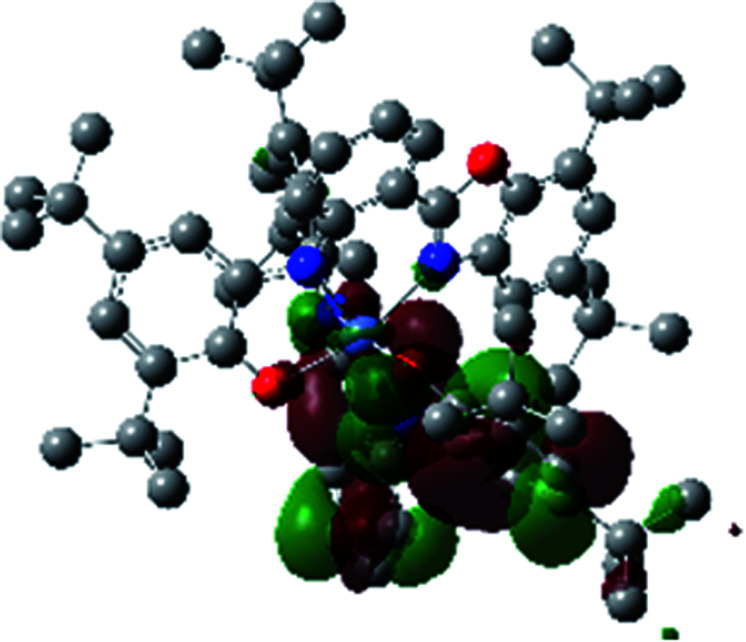

### Catalytic activity of Ni^II^L_2_^BIS^ and Ni^II^L_2_^NIS^ in A^3^-coupling reactions

**Scheme 7:**
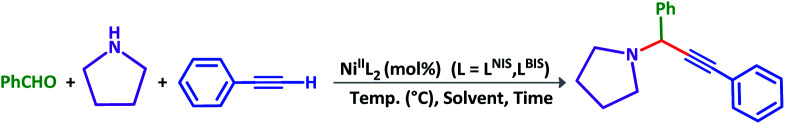
General reaction for testing the catalytic activity of Ni^II^L_2_^BIS^ and Ni^II^L_2_^NIS^ in A^3^-coupling reactions.

General reaction of catalytic activity of Ni^II^L_2_^BIS^ and Ni^II^L_2_^NIS^ in A^3^-coupling reactions is shown in Scheme 7. This reaction was optimized for the various parameters such as temperature, solvent and catalyst loading.

The reaction temperature was initially optimized by performing the model reaction of benzaldehyde, pyrrolidine and phenylacetylene under solvent-free conditions at different temperatures (Table 7 entries 1–5). The model reaction was screened at RT in the presence of 2 mol% NiL_2_^NIS^ catalyst, but a good yield was not obtained during 5 h. As the reaction temperature increased, the reaction time decreased, and the best result was obtained at 85 °C. With increasing temperature up to the optimized temperature (85 °C), a decrease in the yield of desired product was observed, that is related to the evaporation of the volatile precursors from the reaction media in solvent free conditions and also increasing by-products of the reaction.

**Table 7:** Optimization of conditions for the NiL_2_^NIS^ and NiL_2_^BIS^ catalytic activity in A^3^-coupling reaction^a^

Entry	Catalyst	Temp. (°C)	Catalyst amount	Solvent	Time (h)	Yield (%)
1	NiL_2_^NIS^	RT	2 mol%	—	5	35
2	NiL_2_^NIS^	50	2 mol%	—	3	43
3	NiL_2_^NIS^	70	2 mol%			57
**4**	NiL_2_^NIS^	**85**	**2 mol%**	**—**	**2**	**86**
5	NiL_2_^NIS^	100	2 mol%	—	2	80
6	NiL_2_^NIS^	85	1 mol%	—	3	67
7	NiL_2_^NIS^	85	3 mol%	—	2	83
8	Catalyst-free	85	—	—	10	NR
9	NiL_2_^NIS^	100	2 mol%	PhCH_3_	4	—
10	NiL_2_^NIS^	80, reflux	2 mol%	CH_3_CN	4	—
11	NiL_2_^NIS^	65, reflux	2 mol%	THF	2	54
12	NiL_2_^BIS^	80	2 mol%	—	3	—
13	NiL_2_^BIS^	90	3 mol%	—	4	11
14	NiL_2_^BIS^	100	3 mol%	—	4	15
15	NiL_2_^BIS^	100	4 mol%	—	4	24
16	NiL_2_^BIS^	65, reflux	4 mol%	THF	4	10

a All reactions were carried out with benzaldehyde (1 mmol), pyrrolidine (1.1 mmol), phenylacetylene (1.2 mmol).

To optimize the catalyst load, the model reaction was performed in the presence of various amounts of the catalyst 1–3 mol%. According to the results, 2 mol% NiL_2_^NIS^ catalyst shows the best efficiency (Table 7, entries 4, 6–8). The effect of various solvents was also monitored by performing the model reaction in the presence of 2 mol% NiL_2_^NIS^ catalyst (Table 7, entries 9–11).

Then we tried to investigate the A^3^-coupling reaction for the other Ni^II^ complex, NiL_2_^BIS^. It is noteworthy to mention that, this reaction did not happen in any measurable amount (Table 7, entries 12–16), which is due to the lack of free space in the coordination sphere of the six-coordinated NiL_2_^BIS^ complex.

It other words, in the solution medium this complex keeps its stability and departure of ligands does not occur.

Also, a catalyst recycle experiment (Table 8) was done. The catalyst was recovered by centrifugation and the reaction was carried out for three cycles with a slight decrease in activity.

**Table 8:** Recycling of the catalytic system in A^3^ coupling of benzaldehyde, pyrrolidine and phenylacetylene^a^

Run	Time (h)	Yield (%)
1	2	86
2	2	81
3	2	70

a Reaction conditions: benzaldehyde (1 mmol), pyrrolidine (1.1 mmol), phenylacetylene (1.2 mmol), NiL_2_^NIS^ (2 mol%), solvent free, 85 °C.

Encouraged by the optimization results, we turned our attention to various aldehydes and amines. Interestingly, various aldehydes reacted effectively with pyrrolidine, morpholine and piperidine.

As exemplified in Table 9, this protocol is rather general for a wide variety of electron-rich as well as electron-deficient aromatic aldehydes and also various secondary amines. Results show the reaction was performed faster for the aldehydes bearing the electron-withdrawing group such as –NO_2_. In addition, pyrrolidine has the highest activity among the used amines (Table 9, entries e and g).

**Table 9:** NiL_2_^NIS^ catalyzed synthesis of propargylamine derivatives through A^3^-coupling^a^

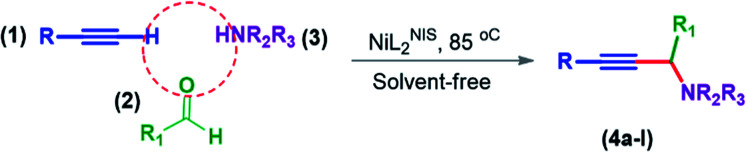									
	Substrate (2)	Product (4)	Time^b^	Yield^c^		Substrate (2)	Product (4)	Time^b^	Yield^c^
	Substrate (3)					Substrate (3)			
a	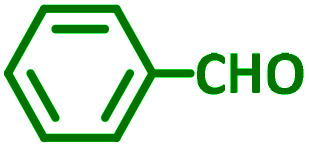	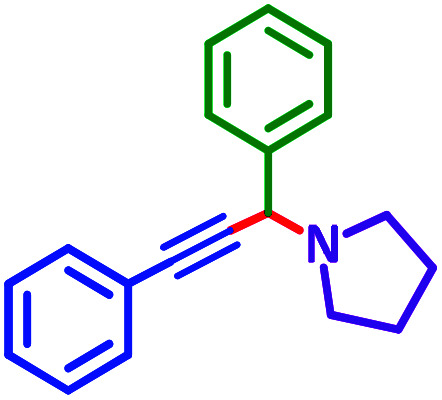	2 h	86%	g	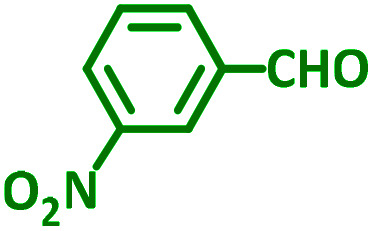	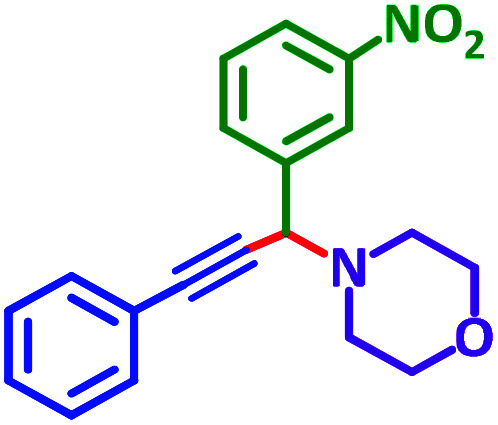	2 h	78%
	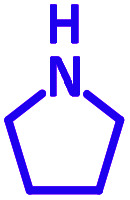					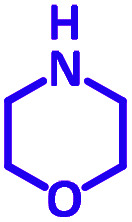			
b	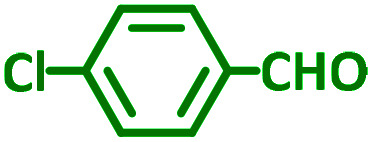	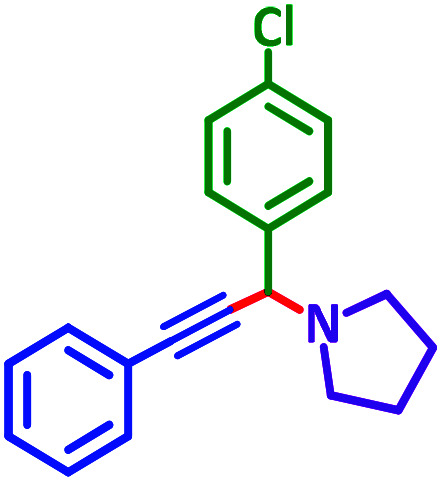	2 h	81%	h	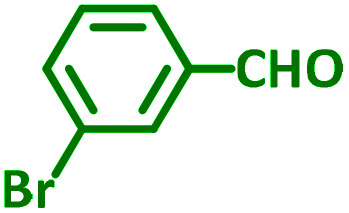	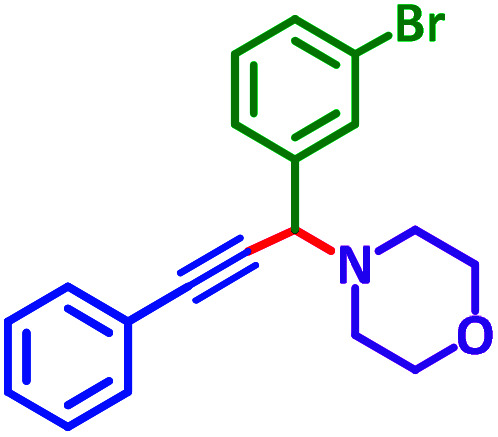	4 h	80%
	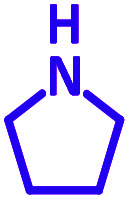					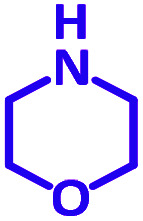			
c	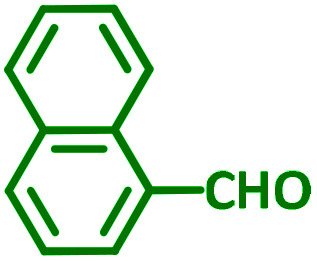	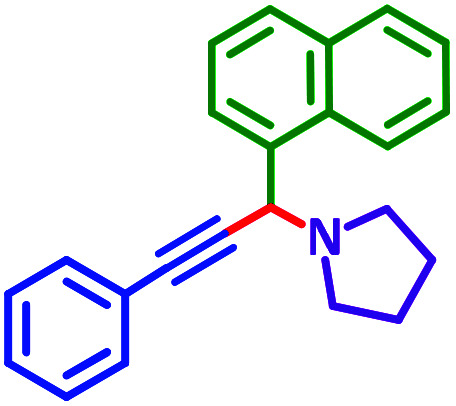	3 h	83%	i	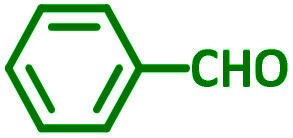	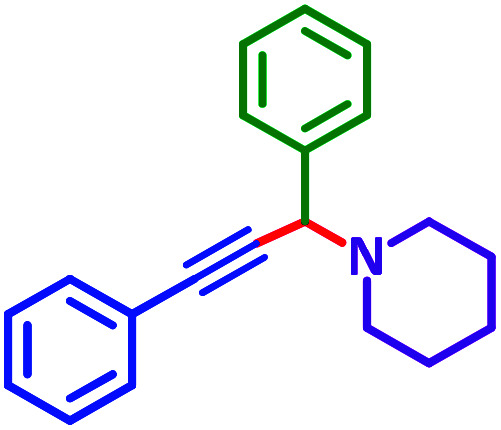	3 h	84%
	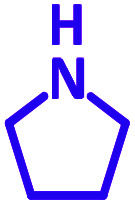					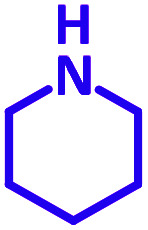			
d	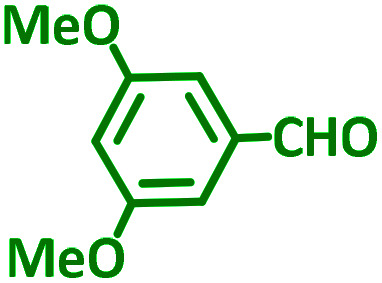	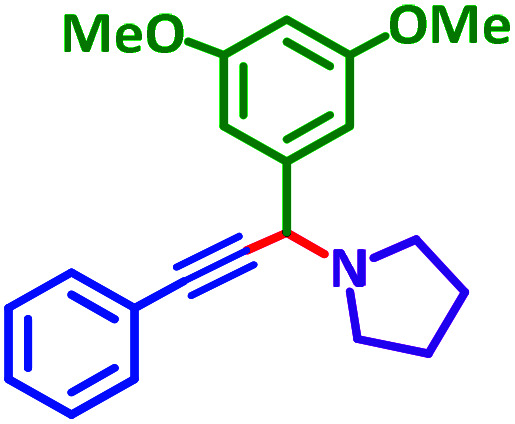	3 h	88%	j	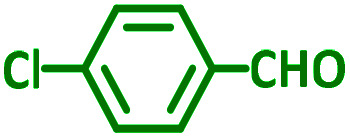	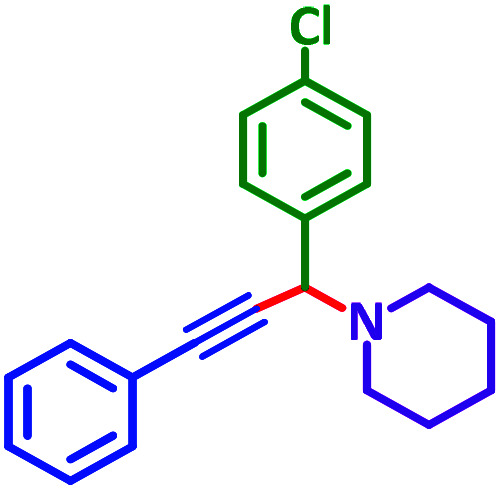	3 h	86%
	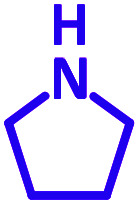					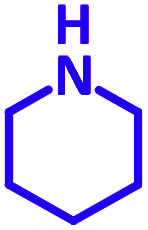			
e	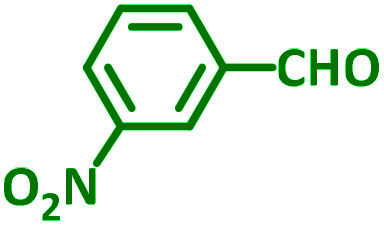	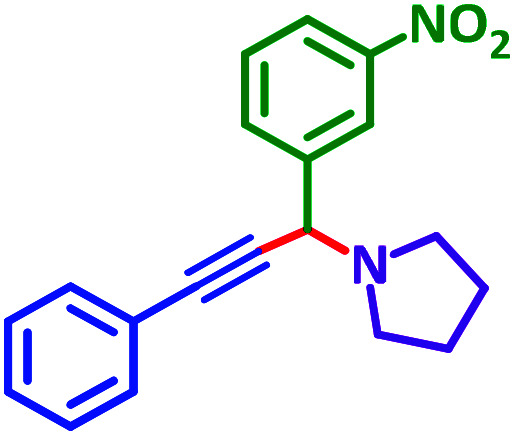	1 h	75%	k	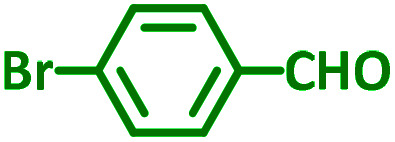	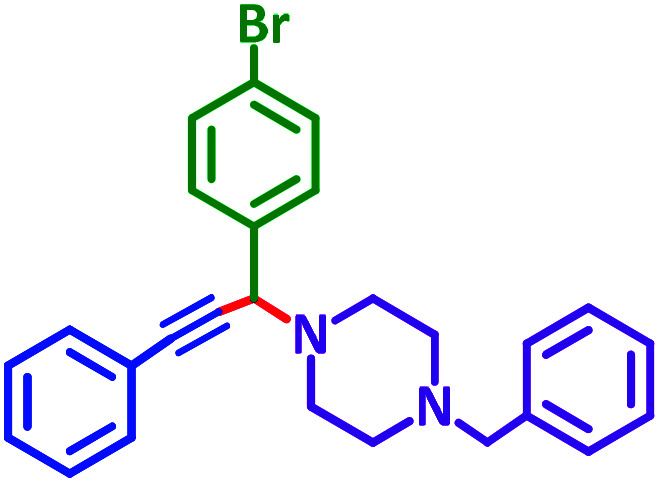	4 h	86%
	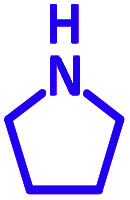					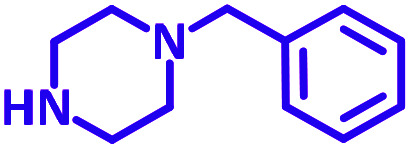			
f	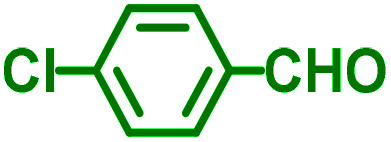	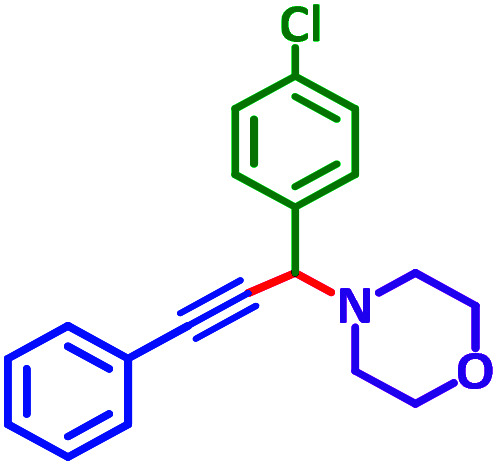	3.5 h	79%	l	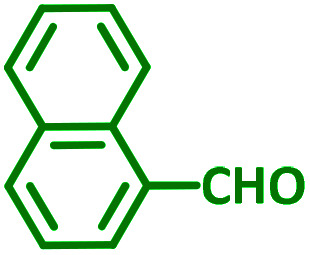	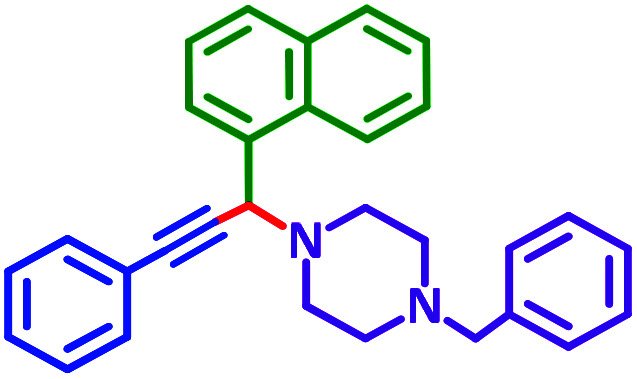	4 h	88%
	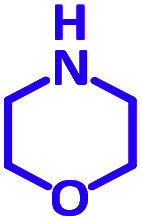					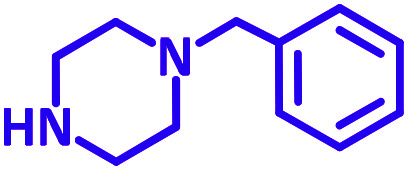			

a Reaction conditions: aldehyde (1 mmol), phenylacetylene (1.2 mmol), amine (1.1 mmol), NiL_2_^NIS^ (2 mol%) at 85 °C under solvent-free conditions.

b Reactions time is based on the consumption of aldehyde monitored by TLC.

c Isolated yield.

Then we tried to compare the activity of our catalyst, NiL_2_^NIS^, and other catalysts which had been studied for A^3^-coupling in the literature (Table 10).^17*a*–*g*^ To the best of our knowledge, there is only one more report of the Ni-catalyzed three-component coupling of aldehyde, alkyne and amine (Table 10, entry 1).

**Table 10:** Reaction conditions for the A^3^-coupling catalyzed with some catalysts

Catalyst (amount)	Reaction condition: *T* (h)/temp. °C/solvent	AA (%)	Ref.
Ni–Y zeolite (20 mg)	4/80/—	97	17*a*
Cyclohexanecarbaldehyde/morpholine/phenylacetylene			
Cu^I^_2_(pip)_2_^a^ (0.4 mol%)	2/110/toluene	86	17*b*
Pyrrolidine/benzaldehyde/phenylacetylene			
Ag/Ni-MOF (0.3 mol%) N_2_ (1 atm)	30 min/80/MeCN	93	17*c*
Pyrrolidine/aldehyde/phenylacetylene			
CuI (10 mol%)	12/100/PEG-400	92	17*d*
Pyrrolidine/benzaldehyde/phenylacetylene			
Cu(OTF)_2_ (10 mol%)	6/80/toluene	92	17*e*
Pyrrolidine/methyl aldehyde/n-hexynyle			
Au NPs (10 mol%)	5/75/ACN	97	17*f*
Piperidine/benzaldehyde/phenylacetylene			
CuCl/succinic acid (20 mol%)	3/50/DCM	85	17*g*
Piperidine/benzaldehyde/1-phenyl-2-trimethylsilylacetylene			
NiL_2_^NIS^**(2 mol%)**	**2/85/—**	**86**	**This work**
**Pyrrolidine/benzaldehyde/phenylacetylene**			

a (pip = (2-picolyliminomethyl)pyrrole anion).

Also, the mechanism involved in our Ni complex-catalyzed A^3^-coupling reaction can be different from the reaction mechanism which is dominant in other complex-catalyzed cross-coupling reactions due to the ability of NiL_2_^NIS^ to present radical species.

Both Ni^0^ and Ni^II^ species are used as Ni sources in Ni-catalyzed cross-couplings and Ni^0^ sources are generally considered as the catalytically active ones. While the easiest way is to use Ni^0^ reagents like Ni-(COD)_2_ and Ni(PPh_3_)_4_,^18^ these nickel sources are difficult to handle because of the high air sensitivity and thermal instability. Alternatively, Ni^II^ complexes are more convenient as pre-catalysts in terms of their availability and easy handling. Nevertheless, these Ni^II^ catalysts should be activated *in situ* with some additives^19^ such as base, I_2_ or PPh_3_ or used as bimetallic systems of Ni(ii)/M(Zn(0), Mn(0), Cu(i), Ag(i). In some cases, Ni complex itself can act as the active catalyst (Table 10, entry 1)^19*a*^ by cleavage of one of the oxo bridges of the ligand and generating an initial nickel(ii) acetylide intermediate in A^3^-coupling reactions. Also, our Ni complex NiL_2_^NIS^ can easily undergo a switch among the different oxidation states of the ligand easily. This event supports the non-innocent behavior of *o*-aminophenol ligand that can act as an “electron reservoir” and accept and donate electrons in a reverse catalytic cycle, result in a high reactivity. This behavior of NiL_2_^NIS^ is well utilized in the homo-coupling of phenyl acetylene.^1^

**Scheme 8:**
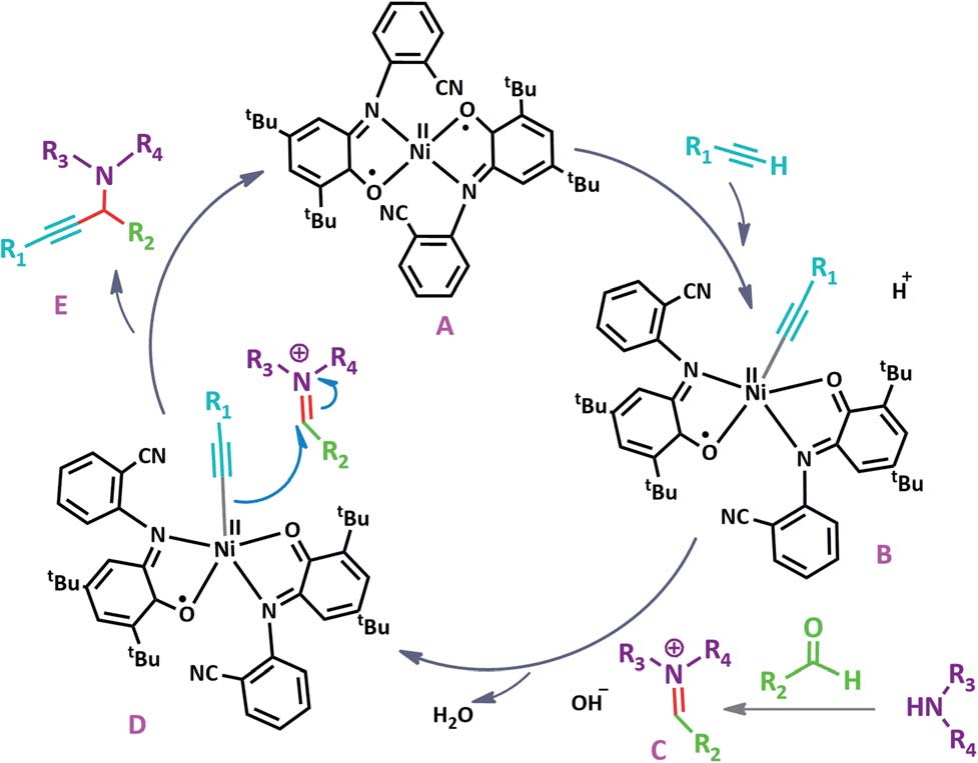
Proposed reaction mechanism.

A plausible reaction pathway is proposed as shown in Scheme 8. In the present work NiL_2_^NIS^ itself acts as the active catalyst generating an initial nickel(ii) acetylide intermediate B by the reaction between a Ni(ii) center in the NiL_2_^NIS^ complex with a terminal alkyne. One of the non-innocent iminosemiquinone L^NIS^ ligands undergoes changes in its oxidation state and iminosemiquinonate/iminoquinonate form of the Ni^II^ complex, [Ni^II^L^NIS^L^NIQ^]^+^, (B), is achieved and keeps the total oxidation state unchanged. Moreover, complexation of this nickel acetylide intermediate with iminium ion, C, which is generated *in situ* from the aldehyde and amine, provides complex D and Ni acetylide complex coordinated with an iminium ion. Finally, the addition of acetylide to the iminium ion within the coordination sphere of nickel(ii) gives propargylamine product E and regenerates the nickel catalyst A for the subsequent reactions.

## Experimental

### Material and method

All reagents were acquired *via* commercial sources unless stated otherwise. 3,5-DTBQ (3,5-di-*tert*-butylcyclohexa-3,5-diene-1,2-dione) was synthesized from the procedure reported in literature.^20^ The ligand H_2_L^BAP^ was synthesized following the reported procedure.^6*a*^

Elemental analyses (C, H, and N) were done by the Elementar, Vario EL III. Fourier transform infrared spectroscopy with KBr pellets was performed on a FT IR Bruker Vector 22 instrument. NMR spectra were performed at 400 MHz on a Bruker DRX spectrometer in CDCl_3_ solution. UV-Vis absorbance spectra was recorded by using a CARY 100 Bio spectrophotometer. Cyclic voltammetry (CV) was carried out on a PAR-263A potentiometer. The cell was prepared with an Ag wire reference electrode, a glassy carbon working electrode, and a Pt counter electrode with 0.1 M NBu_4_ClO_4_ (TBAP) solutions in CH_2_Cl_2_. Ferrocene was used as an internal standard. The magnetic measurements were achieved with the use of a Quantum Design SQUID magnetometer MPMS-XL between 1.8 and 290 K with a dc applied field of 1000 Oe. Measurement was done on polycrystalline sample of 35 mg for NiL_2_^BIS^. Sample for X-band measurement was placed in 4 mm outer-diameter sample tubes with sample volumes of ∼300 μL.

All catalytic reactions were monitored by TLC (thin layer chromatography) and all yields refer to isolated products. ^1^H NMR spectra were recorded in CDCl_3_ on a Bruker DRX-400 AVANCE (400 MHz for ^1^H and 100 MHz for ^13^C) spectrometer.

### Synthesis

Ni^II^L_2_^BIS^: synthesis of the ligand (H_2_L^BAP^), 2,4-di-*tert*-butyl-6-(2-(5,7-di-*tert*-butylbenzo [d] oxazol-2-yl) phenylamino) phenol, used in this work has previously been reported by our group.^6*a*^

To a magnetically stirred mixture of H_2_L^BAP^ (0.524 g; 1 mmol) and Et_3_N in acetonitrile (5 mL), Ni^II^(OAc)_2_·4H_2_O (0.248 g, 1 mmol) was added drop-wise and the resulting mixture was stirred for 3 h. The dark green precipitate that formed was collected by filtration, washed with cold MeOH and single crystals of suitable dimensions for X-ray analysis obtained from recrystallization of a concentrated solution of the microcrystalline solid in MeOH–CH_2_Cl_2_ (2 : 1; v/v) mixture. Yield: (78%). Anal. Calcd (%) for C_49_H_68_NiN_3_O_4_: C 74.02 (74.93), H 8.51 (8.81), N 4.83 (4.86). *ν*_max_(KBr)/cm^−1^: 2962 (C–H), 1614 (C=C), 1470 (C=N), 1268 (C–O), 1164 (C–N) (Fig. S1†).

### X-ray crystallography

The X-ray data were collected with the Oxford Sapphire CCD diffractometer, at 293(2) K using MoKα radiation *λ* = 0.71073 Å. The structure was solved by direct methods with SHELXT and refined with the full-matrix least-squares method on F^2^ with the use of SHELX2017 program package.^21^ The analytical absorption correction was applied by CrysAlis 171.38.43 package of programs.^22^ The large voids are found in the structure, covering 18.4% of the unit cell volume, but no significant density peaks have been located there. For the solvent accessible volume 1344 Å^3^, 24 electrons were found. Therefore, to account for the disordered solvent contribution to the structure factors, the SQUEEZE (Version 160617) program was used.^23^ The hydrogen atom positions were determined from the difference maps, and all hydrogen atoms were constrained during refinement. Four *t*Bu groups (C24, C28, C32 and C68) revealed the rotational disorder. The attempt to define the discrete odel forthat disorder has given no improvement in the model quality. Therefore, the SIMU constraints for the disordered *t*Bu groups and ISOR constraint the C25 methyl group were used in the final refinement. A summary of the crystal data and refinement details for compound 1 are given in Table 1. The structural data have been deposited at the Cambridge Crystallographic Data Center: (CCDC no. 2035886).

### Computational details

Geometry optimization was performed on the Ni complex using the hybrid B3LYP method, with LANL2DZ basis set for nickel and 6-31G* basis set for all other atoms, using the program Gaussian 16.^24^

### General procedure for the synthesis of propargylamine derivatives

In a typical reaction benzaldehyde (1 mmol), pyrrolidine (1.1 mmol), phenylacetylene (1.2 mmol) and catalyst, Ni^II^L_2_^NIS^, (2 mol%, 19 mg) were added into a 5 mL round bottom flask and were stirred at 85 °C. The progress of the reaction was monitored by TLC. After completion of the reaction, *n*-hexane (2 mL) was added to the reaction mixture and the solution was centrifuged at 3000 rpm for 2 min. Finally, the excess of solvent was removed under reduced pressure to give the corresponding product. Further purification was achieved by thin layer chromatography on silica gel using *n*-hexane/ethyl acetate. Then, the recovered catalyst was reused in recycle experiment of A^3^ coupling reaction of benzaldehyde, pyrrolidine and phenylacetylene. Physical and spectroscopic data for selected compounds are given in Fig. S3 to S16†).

## Conclusion

Complex Ni^II^L_2_^BIS^ was synthesized and characterized in the current work. A combination of experimental and theoretical studies is used to investigate the electronic properties of this nickel complex. The synthesis of Ni^II^L_2_^BIS^ neutral complex was achieved by ligation of the *o*-iminosemiquinone 1-electron oxidized form of the tridentate *o*-aminophenol benzoxazole-based ligand H_2_L^BAP^ and characterized by X-ray crystallography. The bond lengths of the L^BIS^ ligand indicate that is found in the semiquinone form. This observation is also supported by the electronic configuration as determined by density functional theory calculations.

Then the catalytic activity of this complex, NiL_2_^BIS^, and the previously reported complex, NiL_2_^NIS^^1^ in three-component coupling of aldehydes, amines and alkynes (A^3^-coupling) was investigated and the four-coordinated NiL_2_^NIS^ complex was found to be significantly more efficient catalyst due to the non-innocent *o*-aminophenol ligand that acts as an “electron reservoir” and can accept and donate electrons in the C–H activation stage of catalytic cycle resulting a high reactivity in A^3^-coupling reaction. This procedure is also environmentally friendly and is done under solvent-free conditions as it does not require any organic solvents. Good yields and mild reaction conditions are other remarkable advantages of this process. The catalyst can be readily recovered and reused for three cycles with a slight decrease in activity.

## Conflicts of interest

There are no conflicts to declare.

## Supplementary Material

RA-011-D0RA10248B-s001

RA-011-D0RA10248B-s002
