# Supplementary material for: Biradical o-iminobenzosemiquinonato(1−) complexes of nickel(ii): catalytic activity in three-component coupling of aldehydes, amines and alkynes
Source: RSC Adv. 2021 Apr 6;11(21):12845–59. doi: 10.1039/d0ra10248b (PMC8697240; doi:10.1039/d0ra10248b)
Supplement: RA-011-D0RA10248B-s001 [file RA-011-D0RA10248B-s001.pdf]

Electronic Supplementary Material (ESI) for RSC Advances.
This journal is © The Royal Society of Chemistry 2021

Biradical *o*-Iminobenzosemiquinonato(1−) Complexes of Nickel(II): Catalytic Activity in Three-Component Coupling of Aldehydes, Amines and Alkynes

Mina Nasibipour [a], Elham Safaei [a,*], Ali Moaddeli [a], Marziyeh Sadat Masoumpour [b], Andrzej Wojtczak [c]

[a] *Department of Chemistry, College of Sciences, Shiraz University, 71454, Shiraz, Iran*
[b] *Department of Chemistry, Estahban Higher Education Center, Estahban 74519-44655, Iran*
[c] *Nicolaus Copernicus University, Faculty of Chemistry, 87-100 Torun, Poland*

Contents	Page
**Figure S1** IR spectrum of $Ni^{II}L_2^{BIS}$.	S2
**Table S1** Crystallographic data and structure refinement of $Ni^{II}L_2^{BIS}$.	S2
**Table S2** Bond distances (Å) and bond angles (°) $Ni^{II}L_2^{BIS}$.	S3
**Table S3** Torsion angles [°] for $Ni^{II}L_2^{NIS}$.	S6
**Table S4** Selected bond lengths of optimized structure of $Ni^{II}L_2^{BIS}$.	S9
**Figure S2** Absorption and emission spectrums of the 0.1mM CH_2Cl_2 solutions of H_2L^{BAP} ligand.	S10
**Figure S3** ^{1}H NMR and ^{13}C NMR of 2.5.1 1-(1-(Naphthalen-1-yl)-3-phenylprop-2-yn-1-yl)pyrrolidine.	S10
**Figure S4** IR spectrum of 2.5.1 1-(1-(Naphthalen-1-yl)-3-phenylprop-2-yn-1-yl)pyrrolidine.	S10
**Figure S5** ^{1}H NMR of 1-(1-(3,5-Dimethoxyphenyl)-3-phenylprop-2-yn-1-yl)pyrrolidine.	S11
**Figure S6** IR spectrum of 1-(1-(3,5-Dimethoxyphenyl)-3-phenylprop-2-yn-1-yl)pyrrolidine.	S11
**Figure S7** ^{1}H NMR and ^{13}C NMR of 4-(1-(4-Chlorophenyl)-3-phenylprop-2-yn-1-yl)morpholine.	S12
**Figure S8** IR spectrum of 4-(1-(4-Chlorophenyl)-3-phenylprop-2-yn-1-yl)morpholine.	S12
**Figure S9** ^{1}H NMR and ^{13}C NMR of 1-Benzyl-4-(1-(naphthalen-1-yl)-3-phenylprop-2-yn-1-yl) piperazine.	S13
**Figure S10** IR spectrum of 1-Benzyl-4-(1-(naphthalen-1-yl)-3-phenylprop-2-yn-1-yl) piperazine.	S13
**Figure S11** ^{1}H NMR of 1-benzyl-4-(1-(4-bromophenyl)-3-phenylprop-2-yn-1-yl)piperazine.	S14
**Figure S12** IR spectrum of 1-(1,3-diphenylprop-2-yn-1-yl)pyrrolidine.	S14
**Figure S13** IR spectrum of 1-(1-(3-nitrophenyl)-3-phenylprop-2-yn-1-yl)morpholine.	S14
**Figure S14** IR spectrum of 4-(1-(4-Bromophenyl)-3-phenylprop-2-yn-1-yl)morpholine.	S15
**Figure S15** IR spectrum of 1-(1,3-diphenylprop-2-yn-1-yl)Piperidine.	S15
**Figure S16** IR spectrum of 4-(1-(4-Chlorophenyl)-3-phenylprop-2-yn-1-yl)Piperidine.	S15

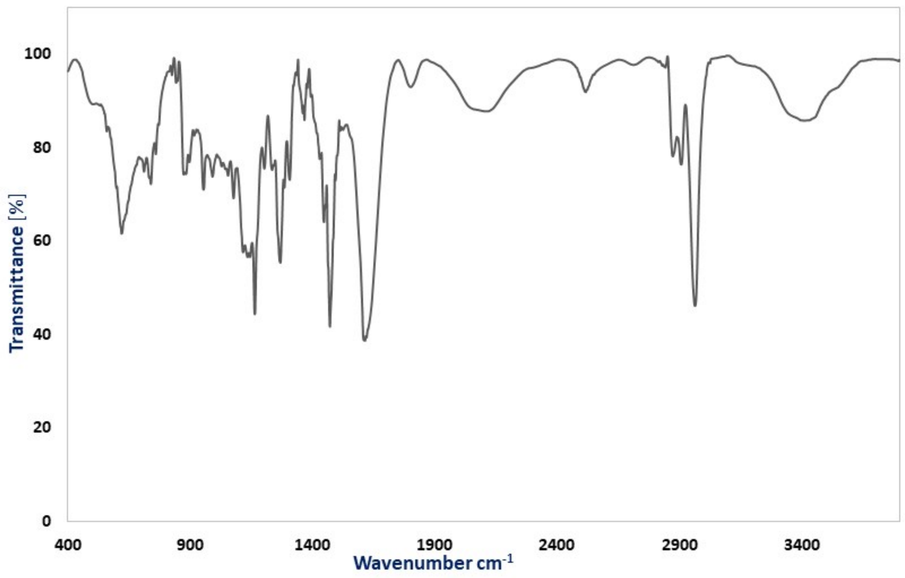

Figure S1 IR spectrum of $Ni^{II}L_2^{BIS}$.

Table S1 Crystal data and structure refinement for $Ni^{II}L_2^{BIS}$.

Identification code	e1464a_sq
Empirical formula	C70 H88 N4 Ni O4
Formula weight	1108.15
Temperature; K	293(2)
Wavelength; Å	0.71073
Crystal system	Monoclinic
Space group	$P2_1/n$
Unit cell dimensions; Å, °	a = 18.1423(6)
	b = 17.5195(5)
	c = 23.9371(9)
	α = 90
	β = 106.001(3)
	γ = 90
Volume; $Å^3$	7313.5(4)
Z	4
Density (calculated); Mg/m^3	1.006
Absorption coefficient; mm^{-1}	0.308
F(000)	2384
Crystal size; mm	0.793 x 0.564 x 0.517
Theta range for data collection	2.325 to 28.366°.
Index ranges	-22<=h<=21, -22<=k<=22, -31<=l<=29
Reflections collected	52213
Independent reflections	16548 [R(int) = 0.0442]
Completeness to theta = 25.242°	99.9 %
Absorption correction	Analytical
Max. and min. transmission	0.883 and 0.839
Refinement method	Full-matrix least-squares on F^2
Data / restraints / parameters	16548 / 0 / 712
Goodness-of-fit on F^2	1.040
Final R indices [I>2sigma(I)]	R1 = 0.0614, wR2 = 0.1426
R indices (all data)	R1 = 0.1017, wR2 = 0.1725
Largest diff. peak and hole; e.$Å^{-3}$	0.649 and -0.271

Table 2 Bond lengths [Å] and angles [°] for $Ni^{II}L_2^{BIS}$.

Ni(1)-N(41)	2.0193(19)
Ni(1)-N(1)	2.027(2)
Ni(1)-O(41)	2.0425(18)
Ni(1)-O(1)	2.0466(19)
Ni(1)-N(2)	2.143(2)
Ni(1)-N(42)	2.153(2)
O(1)-C(1)	1.286(3)
C(1)-C(2)	1.446(4)
C(1)-C(6)	1.466(4)
C(2)-C(3)	1.375(4)
C(2)-C(20)	1.536(5)
C(3)-C(4)	1.427(5)
C(4)-C(5)	1.368(5)
C(4)-C(24)	1.547(4)
C(5)-C(6)	1.428(4)
C(6)-N(1)	1.355(4)
N(1)-C(7)	1.398(4)
C(7)-C(8)	1.412(4)
C(7)-C(12)	1.414(4)
C(8)-C(9)	1.376(5)
C(9)-C(10)	1.371(6)
C(10)-C(11)	1.388(5)
C(11)-C(12)	1.395(4)
C(12)-C(13)	1.469(4)
C(13)-N(2)	1.302(3)
C(13)-O(2)	1.372(3)
N(2)-C(14)	1.411(3)
C(14)-C(19)	1.381(4)
C(14)-C(15)	1.396(4)
C(15)-C(16)	1.395(4)
C(16)-C(17)	1.405(4)
C(16)-C(28)	1.548(4)
C(17)-C(18)	1.397(4)
C(18)-C(19)	1.400(4)
C(18)-C(32)	1.521(4)
C(19)-O(2)	1.392(3)
C(20)-C(21)	1.530(6)
C(20)-C(23)	1.551(5)
C(20)-C(22)	1.556(5)
C(24)-C(25)	1.461(7)
C(24)-C(27)	1.474(8)
C(24)-C(26)	1.575(8)
C(28)-C(29)	1.448(7)
C(28)-C(30)	1.463(6)
C(28)-C(31)	1.527(8)
C(32)-C(35)	1.513(5)
C(32)-C(33)	1.523(6)
C(32)-C(34)	1.551(5)
O(41)-C(41)	1.283(3)
C(41)-C(42)	1.452(4)
C(41)-C(46)	1.465(3)
C(42)-C(43)	1.369(4)
C(42)-C(60)	1.546(4)
C(43)-C(44)	1.430(4)
C(44)-C(45)	1.375(4)
C(44)-C(64)	1.536(4)
C(45)-C(46)	1.420(3)
C(46)-N(41)	1.363(3)
N(41)-C(47)	1.394(3)
C(47)-C(48)	1.401(4)
C(47)-C(52)	1.418(3)
C(48)-C(49)	1.386(4)
C(49)-C(50)	1.384(5)
C(50)-C(51)	1.377(4)
C(51)-C(52)	1.405(4)
C(52)-C(53)	1.465(3)
C(53)-N(42)	1.306(3)
C(53)-O(42)	1.371(3)
N(42)-C(54)	1.415(3)
C(54)-C(59)	1.382(4)
C(54)-C(55)	1.392(4)
C(55)-C(56)	1.396(4)

C(56)-C(57)	1.405(4)
C(56)-C(68)	1.547(4)
C(57)-C(58)	1.396(4)
C(58)-C(59)	1.397(4)
C(58)-C(72)	1.535(4)
C(59)-O(42)	1.392(3)
C(60)-C(62)	1.536(6)
C(60)-C(61)	1.543(6)
C(60)-C(63)	1.546(5)
C(64)-C(65)	1.536(5)
C(64)-C(67)	1.538(5)
C(64)-C(66)	1.542(5)
C(68)-C(69)	1.504(6)
C(68)-C(70)	1.509(7)
C(68)-C(71)	1.517(7)
C(72)-C(74)	1.525(6)
C(72)-C(75)	1.527(6)
C(72)-C(73)	1.540(5)
N(41)-Ni(1)-N(1)	171.41(9)
N(41)-Ni(1)-O(41)	80.73(7)
N(1)-Ni(1)-O(41)	93.90(8)
N(41)-Ni(1)-O(1)	94.40(8)
N(1)-Ni(1)-O(1)	80.16(8)
O(41)-Ni(1)-O(1)	102.55(8)
N(41)-Ni(1)-N(2)	98.03(8)
N(1)-Ni(1)-N(2)	88.61(9)
O(41)-Ni(1)-N(2)	89.99(8)
O(1)-Ni(1)-N(2)	163.62(8)
N(41)-Ni(1)-N(42)	88.14(8)
N(1)-Ni(1)-N(42)	98.28(8)
O(41)-Ni(1)-N(42)	164.88(8)
O(1)-Ni(1)-N(42)	88.40(8)
N(2)-Ni(1)-N(42)	81.41(8)
C(1)-O(1)-Ni(1)	111.03(17)
O(1)-C(1)-C(2)	122.8(3)
O(1)-C(1)-C(6)	117.5(2)
C(2)-C(1)-C(6)	119.7(2)
C(3)-C(2)-C(1)	116.8(3)
C(3)-C(2)-C(20)	123.8(3)
C(1)-C(2)-C(20)	119.4(3)
C(2)-C(3)-C(4)	124.9(3)
C(5)-C(4)-C(3)	118.6(3)
C(5)-C(4)-C(24)	121.3(4)
C(3)-C(4)-C(24)	120.1(4)
C(4)-C(5)-C(6)	121.2(3)
N(1)-C(6)-C(5)	126.6(3)
N(1)-C(6)-C(1)	114.3(2)
C(5)-C(6)-C(1)	118.8(3)
C(6)-N(1)-C(7)	123.3(2)
C(6)-N(1)-Ni(1)	111.56(17)
C(7)-N(1)-Ni(1)	124.81(19)
N(1)-C(7)-C(8)	122.6(3)
N(1)-C(7)-C(12)	120.1(2)
C(8)-C(7)-C(12)	117.1(3)
C(9)-C(8)-C(7)	121.2(4)
C(10)-C(9)-C(8)	121.3(3)
C(9)-C(10)-C(11)	119.3(4)
C(10)-C(11)-C(12)	120.7(4)
C(11)-C(12)-C(7)	120.4(3)
C(11)-C(12)-C(13)	118.0(3)
C(7)-C(12)-C(13)	121.6(2)
N(2)-C(13)-O(2)	114.4(2)
N(2)-C(13)-C(12)	128.6(3)
O(2)-C(13)-C(12)	117.0(2)
C(13)-N(2)-C(14)	105.1(2)
C(13)-N(2)-Ni(1)	119.78(18)
C(14)-N(2)-Ni(1)	130.06(17)
C(19)-C(14)-C(15)	121.0(3)
C(19)-C(14)-N(2)	108.2(2)
C(15)-C(14)-N(2)	130.8(3)
C(16)-C(15)-C(14)	117.6(3)
C(15)-C(16)-C(17)	119.0(3)
C(15)-C(16)-C(28)	119.7(3)

C(17)-C(16)-C(28)	121.2(3)
C(18)-C(17)-C(16)	125.4(3)
C(17)-C(18)-C(19)	112.6(3)
C(17)-C(18)-C(32)	124.3(3)
C(19)-C(18)-C(32)	123.0(3)
C(14)-C(19)-O(2)	107.8(2)
C(14)-C(19)-C(18)	124.4(3)
O(2)-C(19)-C(18)	127.8(3)
C(13)-O(2)-C(19)	104.4(2)
C(21)-C(20)-C(2)	110.4(3)
C(21)-C(20)-C(23)	108.3(4)
C(2)-C(20)-C(23)	111.8(3)
C(21)-C(20)-C(22)	110.2(3)
C(2)-C(20)-C(22)	108.7(3)
C(23)-C(20)-C(22)	107.4(3)
C(25)-C(24)-C(27)	116.8(6)
C(25)-C(24)-C(4)	111.1(4)
C(27)-C(24)-C(4)	110.1(4)
C(25)-C(24)-C(26)	105.6(6)
C(27)-C(24)-C(26)	101.7(5)
C(4)-C(24)-C(26)	111.1(4)
C(29)-C(28)-C(30)	113.5(6)
C(29)-C(28)-C(31)	106.5(6)
C(30)-C(28)-C(31)	103.9(5)
C(29)-C(28)-C(16)	108.9(4)
C(30)-C(28)-C(16)	113.1(3)
C(31)-C(28)-C(16)	110.7(4)
C(35)-C(32)-C(18)	112.2(3)
C(35)-C(32)-C(33)	110.0(4)
C(18)-C(32)-C(33)	111.0(3)
C(35)-C(32)-C(34)	108.9(3)
C(18)-C(32)-C(34)	107.6(3)
C(33)-C(32)-C(34)	106.9(4)
C(41)-O(41)-Ni(1)	111.27(15)
O(41)-C(41)-C(42)	123.2(2)
O(41)-C(41)-C(46)	118.6(2)
C(42)-C(41)-C(46)	118.2(2)
C(43)-C(42)-C(41)	117.8(3)
C(43)-C(42)-C(60)	123.6(3)
C(41)-C(42)-C(60)	118.6(3)
C(42)-C(43)-C(44)	124.7(3)
C(45)-C(44)-C(43)	118.1(2)
C(45)-C(44)-C(64)	122.5(3)
C(43)-C(44)-C(64)	119.4(2)
C(44)-C(45)-C(46)	121.2(2)
N(41)-C(46)-C(45)	126.3(2)
N(41)-C(46)-C(41)	113.8(2)
C(45)-C(46)-C(41)	119.9(2)
C(46)-N(41)-C(47)	122.0(2)
C(46)-N(41)-Ni(1)	111.79(15)
C(47)-N(41)-Ni(1)	125.67(15)
N(41)-C(47)-C(48)	122.1(2)
N(41)-C(47)-C(52)	120.3(2)
C(48)-C(47)-C(52)	117.5(2)
C(49)-C(48)-C(47)	121.6(3)
C(50)-C(49)-C(48)	120.6(3)
C(51)-C(50)-C(49)	119.1(3)
C(50)-C(51)-C(52)	121.4(3)
C(51)-C(52)-C(47)	119.5(2)
C(51)-C(52)-C(53)	118.3(2)
C(47)-C(52)-C(53)	122.2(2)
N(42)-C(53)-O(42)	114.2(2)
N(42)-C(53)-C(52)	128.8(2)
O(42)-C(53)-C(52)	116.9(2)
C(53)-N(42)-C(54)	105.2(2)
C(53)-N(42)-Ni(1)	120.77(16)
C(54)-N(42)-Ni(1)	131.18(17)
C(59)-C(54)-C(55)	121.0(2)
C(59)-C(54)-N(42)	108.1(2)
C(55)-C(54)-N(42)	130.9(2)
C(54)-C(55)-C(56)	117.8(3)
C(55)-C(56)-C(57)	118.8(3)
C(55)-C(56)-C(68)	121.1(3)

C(57)-C(56)-C(68)	120.1(3)
C(58)-C(57)-C(56)	125.3(3)
C(57)-C(58)-C(59)	112.9(3)
C(57)-C(58)-C(72)	124.7(3)
C(59)-C(58)-C(72)	122.4(3)
C(54)-C(59)-O(42)	107.8(2)
C(54)-C(59)-C(58)	124.2(3)
O(42)-C(59)-C(58)	128.0(3)
C(62)-C(60)-C(61)	110.8(4)
C(62)-C(60)-C(63)	107.3(4)
C(61)-C(60)-C(63)	108.7(4)
C(62)-C(60)-C(42)	110.2(3)
C(61)-C(60)-C(42)	108.6(3)
C(63)-C(60)-C(42)	111.2(3)
C(44)-C(64)-C(65)	111.9(3)
C(44)-C(64)-C(67)	110.5(3)
C(65)-C(64)-C(67)	108.7(3)
C(44)-C(64)-C(66)	108.8(3)
C(65)-C(64)-C(66)	107.9(3)
C(67)-C(64)-C(66)	109.0(3)
C(69)-C(68)-C(70)	108.5(4)
C(69)-C(68)-C(71)	108.0(4)
C(70)-C(68)-C(71)	109.0(5)
C(69)-C(68)-C(56)	111.8(3)
C(70)-C(68)-C(56)	111.7(3)
C(71)-C(68)-C(56)	107.8(4)
C(74)-C(72)-C(75)	109.0(4)
C(74)-C(72)-C(58)	108.5(3)
C(75)-C(72)-C(58)	109.7(3)
C(74)-C(72)-C(73)	109.1(4)
C(75)-C(72)-C(73)	108.8(4)
C(58)-C(72)-C(73)	111.7(3)

Table 3 Torsion angles [°] for $Ni^{II}L_2^{BIS}$.

Ni(1)-O(1)-C(1)-C(2)	159.9(2)
Ni(1)-O(1)-C(1)-C(6)	-20.2(3)
O(1)-C(1)-C(2)-C(3)	-179.1(3)
C(6)-C(1)-C(2)-C(3)	1.0(4)
O(1)-C(1)-C(2)-C(20)	-0.9(4)
C(6)-C(1)-C(2)-C(20)	179.2(3)
C(1)-C(2)-C(3)-C(4)	-0.5(5)
C(20)-C(2)-C(3)-C(4)	-178.6(3)
C(2)-C(3)-C(4)-C(5)	-0.9(5)
C(2)-C(3)-C(4)-C(24)	176.8(3)
C(3)-C(4)-C(5)-C(6)	1.7(5)
C(24)-C(4)-C(5)-C(6)	-176.0(3)
C(4)-C(5)-C(6)-N(1)	172.7(3)
C(4)-C(5)-C(6)-C(1)	-1.2(4)
O(1)-C(1)-C(6)-N(1)	5.3(4)
C(2)-C(1)-C(6)-N(1)	-174.8(2)
O(1)-C(1)-C(6)-C(5)	179.9(2)
C(2)-C(1)-C(6)-C(5)	-0.2(4)
C(5)-C(6)-N(1)-C(7)	24.6(4)
C(1)-C(6)-N(1)-C(7)	-161.4(2)
C(5)-C(6)-N(1)-Ni(1)	-161.3(2)
C(1)-C(6)-N(1)-Ni(1)	12.7(3)
C(6)-N(1)-C(7)-C(8)	34.9(4)
Ni(1)-N(1)-C(7)-C(8)	-138.4(3)
C(6)-N(1)-C(7)-C(12)	-148.8(3)
Ni(1)-N(1)-C(7)-C(12)	37.8(4)
N(1)-C(7)-C(8)-C(9)	178.3(4)
C(12)-C(7)-C(8)-C(9)	1.9(5)
C(7)-C(8)-C(9)-C(10)	0.6(7)
C(8)-C(9)-C(10)-C(11)	-1.9(8)
C(9)-C(10)-C(11)-C(12)	0.5(8)
C(10)-C(11)-C(12)-C(7)	2.1(6)
C(10)-C(11)-C(12)-C(13)	-176.9(4)

N(1)-C(7)-C(12)-C(11)	-179.7(3)
C(8)-C(7)-C(12)-C(11)	-3.3(5)
N(1)-C(7)-C(12)-C(13)	-0.8(4)
C(8)-C(7)-C(12)-C(13)	175.7(3)
C(11)-C(12)-C(13)-N(2)	144.3(3)
C(7)-C(12)-C(13)-N(2)	-34.7(5)
C(11)-C(12)-C(13)-O(2)	-33.3(4)
C(7)-C(12)-C(13)-O(2)	147.7(3)
O(2)-C(13)-N(2)-C(14)	0.8(3)
C(12)-C(13)-N(2)-C(14)	-176.8(3)
O(2)-C(13)-N(2)-Ni(1)	-156.58(18)
C(12)-C(13)-N(2)-Ni(1)	25.8(4)
C(13)-N(2)-C(14)-C(19)	-1.0(3)
Ni(1)-N(2)-C(14)-C(19)	153.10(19)
C(13)-N(2)-C(14)-C(15)	177.2(3)
Ni(1)-N(2)-C(14)-C(15)	-28.6(4)
C(19)-C(14)-C(15)-C(16)	-0.8(4)
N(2)-C(14)-C(15)-C(16)	-178.8(3)
C(14)-C(15)-C(16)-C(17)	-0.6(4)
C(14)-C(15)-C(16)-C(28)	176.2(3)
C(15)-C(16)-C(17)-C(18)	0.7(5)
C(28)-C(16)-C(17)-C(18)	-176.0(3)
C(16)-C(17)-C(18)-C(19)	0.6(4)
C(16)-C(17)-C(18)-C(32)	-179.3(3)
C(15)-C(14)-C(19)-O(2)	-177.5(2)
N(2)-C(14)-C(19)-O(2)	0.9(3)
C(15)-C(14)-C(19)-C(18)	2.3(4)
N(2)-C(14)-C(19)-C(18)	-179.2(3)
C(17)-C(18)-C(19)-C(14)	-2.1(4)
C(32)-C(18)-C(19)-C(14)	177.7(3)
C(17)-C(18)-C(19)-O(2)	177.7(3)
C(32)-C(18)-C(19)-O(2)	-2.4(5)
N(2)-C(13)-O(2)-C(19)	-0.2(3)
C(12)-C(13)-O(2)-C(19)	177.6(2)
C(14)-C(19)-O(2)-C(13)	-0.4(3)
C(18)-C(19)-O(2)-C(13)	179.7(3)
C(3)-C(2)-C(20)-C(21)	-120.1(4)
C(1)-C(2)-C(20)-C(21)	61.9(4)
C(3)-C(2)-C(20)-C(23)	0.6(5)
C(1)-C(2)-C(20)-C(23)	-177.4(3)
C(3)-C(2)-C(20)-C(22)	119.0(4)
C(1)-C(2)-C(20)-C(22)	-59.0(4)
C(5)-C(4)-C(24)-C(25)	-119.0(6)
C(3)-C(4)-C(24)-C(25)	63.5(7)
C(5)-C(4)-C(24)-C(27)	110.1(6)
C(3)-C(4)-C(24)-C(27)	-67.5(6)
C(5)-C(4)-C(24)-C(26)	-1.7(6)
C(3)-C(4)-C(24)-C(26)	-179.3(4)
C(15)-C(16)-C(28)-C(29)	-92.7(6)
C(17)-C(16)-C(28)-C(29)	84.0(7)
C(15)-C(16)-C(28)-C(30)	34.5(6)
C(17)-C(16)-C(28)-C(30)	-148.7(5)
C(15)-C(16)-C(28)-C(31)	150.6(5)
C(17)-C(16)-C(28)-C(31)	-32.7(6)
C(17)-C(18)-C(32)-C(35)	-4.2(5)
C(19)-C(18)-C(32)-C(35)	176.0(4)
C(17)-C(18)-C(32)-C(33)	-127.7(4)
C(19)-C(18)-C(32)-C(33)	52.5(4)
C(17)-C(18)-C(32)-C(34)	115.6(4)
C(19)-C(18)-C(32)-C(34)	-64.2(4)
Ni(1)-O(41)-C(41)-C(42)	166.3(2)
Ni(1)-O(41)-C(41)-C(46)	-15.2(3)
O(41)-C(41)-C(42)-C(43)	-179.9(3)
C(46)-C(41)-C(42)-C(43)	1.6(4)

O(41)-C(41)-C(42)-C(60)	0.8(4)
C(46)-C(41)-C(42)-C(60)	-177.7(3)
C(41)-C(42)-C(43)-C(44)	1.1(5)
C(60)-C(42)-C(43)-C(44)	-179.7(3)
C(42)-C(43)-C(44)-C(45)	-2.5(5)
C(42)-C(43)-C(44)-C(64)	179.0(3)
C(43)-C(44)-C(45)-C(46)	1.1(4)
C(64)-C(44)-C(45)-C(46)	179.5(3)
C(44)-C(45)-C(46)-N(41)	178.1(3)
C(44)-C(45)-C(46)-C(41)	1.5(4)
O(41)-C(41)-C(46)-N(41)	1.6(3)
C(42)-C(41)-C(46)-N(41)	-179.9(2)
O(41)-C(41)-C(46)-C(45)	178.6(2)
C(42)-C(41)-C(46)-C(45)	-2.9(4)
C(45)-C(46)-N(41)-C(47)	24.4(4)
C(41)-C(46)-N(41)-C(47)	-158.8(2)
C(45)-C(46)-N(41)-Ni(1)	-163.6(2)
C(41)-C(46)-N(41)-Ni(1)	13.1(3)
C(46)-N(41)-C(47)-C(48)	34.3(4)
Ni(1)-N(41)-C(47)-C(48)	-136.5(2)
C(46)-N(41)-C(47)-C(52)	-150.5(2)
Ni(1)-N(41)-C(47)-C(52)	38.7(3)
N(41)-C(47)-C(48)-C(49)	178.9(3)
C(52)-C(47)-C(48)-C(49)	3.5(5)
C(47)-C(48)-C(49)-C(50)	1.3(6)
C(48)-C(49)-C(50)-C(51)	-3.3(6)
C(49)-C(50)-C(51)-C(52)	0.3(6)
C(50)-C(51)-C(52)-C(47)	4.6(5)
C(50)-C(51)-C(52)-C(53)	-172.7(3)
N(41)-C(47)-C(52)-C(51)	178.2(2)
C(48)-C(47)-C(52)-C(51)	-6.4(4)
N(41)-C(47)-C(52)-C(53)	-4.6(4)
C(48)-C(47)-C(52)-C(53)	170.9(3)
C(51)-C(52)-C(53)-N(42)	149.1(3)
C(47)-C(52)-C(53)-N(42)	-28.2(4)
C(51)-C(52)-C(53)-O(42)	-26.3(4)
C(47)-C(52)-C(53)-O(42)	156.4(2)
O(42)-C(53)-N(42)-C(54)	0.1(3)
C(52)-C(53)-N(42)-C(54)	-175.4(2)
O(42)-C(53)-N(42)-Ni(1)	-162.83(16)
C(52)-C(53)-N(42)-Ni(1)	21.7(4)
C(53)-N(42)-C(54)-C(59)	-0.3(3)
Ni(1)-N(42)-C(54)-C(59)	160.06(19)
C(53)-N(42)-C(54)-C(55)	178.3(3)
Ni(1)-N(42)-C(54)-C(55)	-21.3(4)
C(59)-C(54)-C(55)-C(56)	-0.7(4)
N(42)-C(54)-C(55)-C(56)	-179.1(3)
C(54)-C(55)-C(56)-C(57)	-0.8(5)
C(54)-C(55)-C(56)-C(68)	176.1(3)
C(55)-C(56)-C(57)-C(58)	1.0(5)
C(68)-C(56)-C(57)-C(58)	-176.0(3)
C(56)-C(57)-C(58)-C(59)	0.3(5)
C(56)-C(57)-C(58)-C(72)	-177.9(3)
C(55)-C(54)-C(59)-O(42)	-178.3(3)
N(42)-C(54)-C(59)-O(42)	0.5(3)
C(55)-C(54)-C(59)-C(58)	2.2(5)
N(42)-C(54)-C(59)-C(58)	-179.1(3)
C(57)-C(58)-C(59)-C(54)	-1.9(4)
C(72)-C(58)-C(59)-C(54)	176.4(3)
C(57)-C(58)-C(59)-O(42)	178.7(3)
C(72)-C(58)-C(59)-O(42)	-3.1(5)
N(42)-C(53)-O(42)-C(59)	0.2(3)
C(52)-C(53)-O(42)-C(59)	176.3(2)
C(54)-C(59)-O(42)-C(53)	-0.4(3)

C(58)-C(59)-O(42)-C(53)	179.1(3)
C(43)-C(42)-C(60)-C(62)	120.8(4)
C(41)-C(42)-C(60)-C(62)	-59.9(4)
C(43)-C(42)-C(60)-C(61)	-117.6(4)
C(41)-C(42)-C(60)-C(61)	61.6(4)
C(43)-C(42)-C(60)-C(63)	2.0(5)
C(41)-C(42)-C(60)-C(63)	-178.8(4)
C(45)-C(44)-C(64)-C(65)	3.5(4)
C(43)-C(44)-C(64)-C(65)	-178.1(3)
C(45)-C(44)-C(64)-C(67)	124.8(4)
C(43)-C(44)-C(64)-C(67)	-56.8(4)
C(45)-C(44)-C(64)-C(66)	-115.6(3)
C(43)-C(44)-C(64)-C(66)	62.8(4)
C(55)-C(56)-C(68)-C(69)	139.1(4)
C(57)-C(56)-C(68)-C(69)	-44.0(5)
C(55)-C(56)-C(68)-C(70)	17.3(6)
C(57)-C(56)-C(68)-C(70)	-165.8(5)
C(55)-C(56)-C(68)-C(71)	-102.4(5)
C(57)-C(56)-C(68)-C(71)	74.5(5)
C(57)-C(58)-C(72)-C(74)	116.2(4)
C(59)-C(58)-C(72)-C(74)	-61.9(5)
C(57)-C(58)-C(72)-C(75)	-124.9(4)
C(59)-C(58)-C(72)-C(75)	57.0(5)
C(57)-C(58)-C(72)-C(73)	-4.2(5)
C(59)-C(58)-C(72)-C(73)	177.8(4)

Symmetry transformations used to generate equivalent atoms

Table S4 Selected bond lengths of optimized structure of $Ni^{II}L_2^{BIS}$.

Ligand 1			Ligand 2		
Bond	Experimental	Theoretical	Bond	Experimental	Theoretical
Ni1-O2	2.046	2.074	Ni1-O85	2.043	2.071
Ni1-N11	2.026	2.084	Ni1-N94	2.019	2.075
Ni1-N23	2.143	2.163	Ni1-N106	2.153	2.160
C3-O2	1.286	1.283	C86-O85	1.283	1.284
C10-N11	1.356	1.360	C93-N94	1.364	1.362
C3-C4	1.447	1.449	C86-C87	1.451	1.449
C4-C5	1.375	1.380	C87-C88	1.370	1.381
C5-C7	1.426	1.432	C88-C90	1.430	1.431
C7-C8	1.368	1.379	C90-C91	1.374	1.380
C8-C10	1.429	1.422	C91-C93	1.420	1.422
C10-C3	1.465	1.468	C93-C86	1.465	1.465

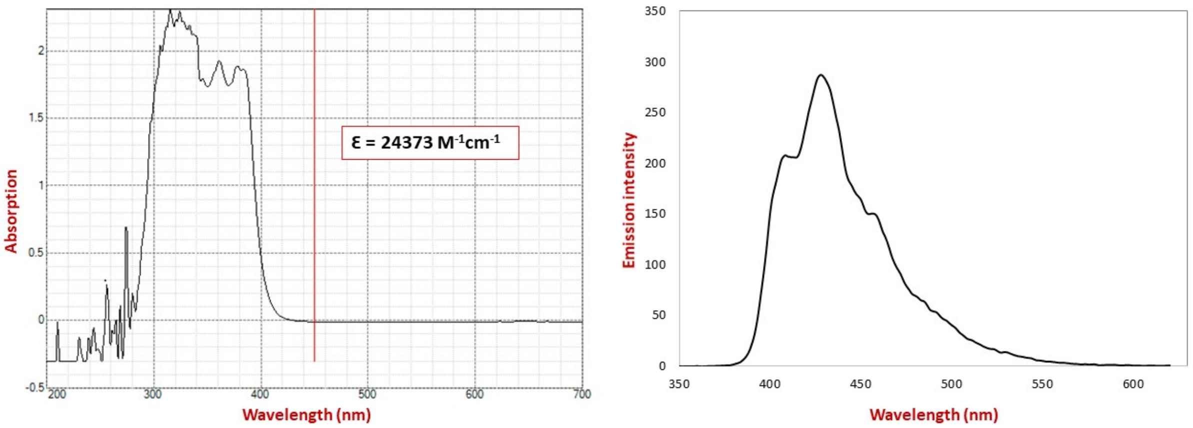

Figure S2 Absorption and emission spectrums of the 0.1mM CH_2Cl_2 solutions of H_2L^{BAP} ligand.

The extinction coefficient value of the H_2L^{BAP} ligand which extracted by the plot of the absorption maxima at a selected wavelength 314 nm versus varied concentrations is 24373 $M^{-1}cm^{-1}$.

Physical and spectroscopic data for selected compounds

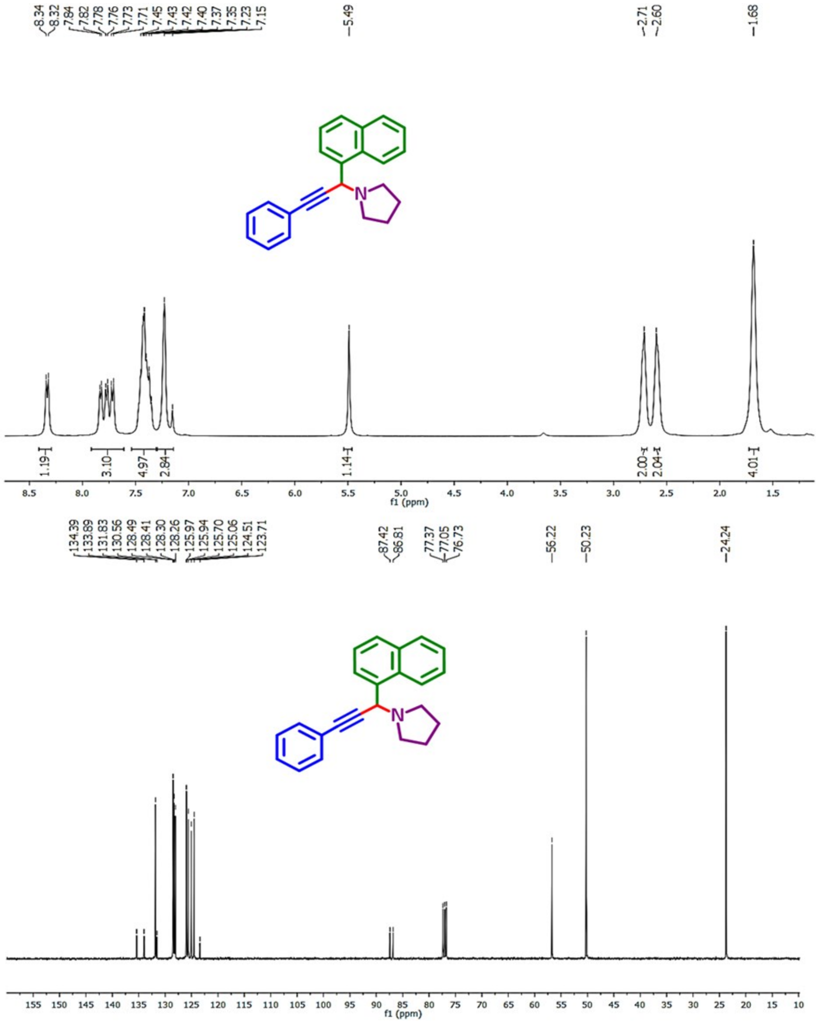

Figure S3 ^{1}H NMR and ^{13}C NMR of 1-(1-(Naphthalen-1-yl)-3-phenylprop-2-yn-1-yl)pyrrolidine.

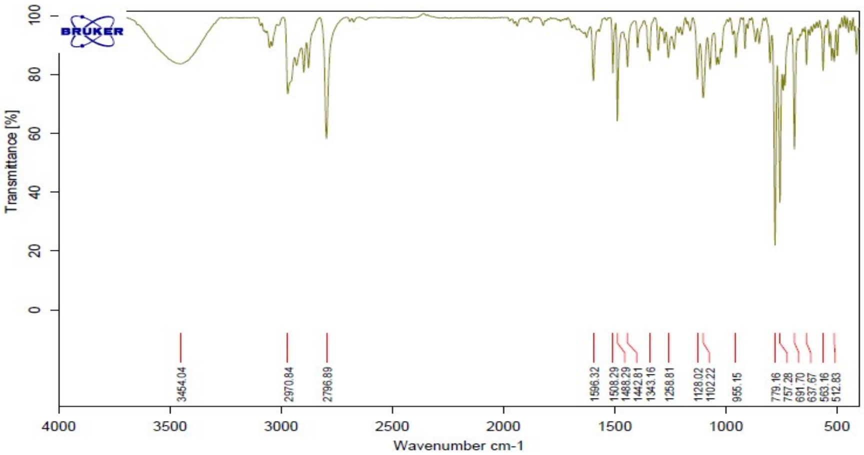

Figure S4 IR spectrum of 2.5.1 1-(1-(Naphthalen-1-yl)-3-phenylprop-2-yn-1-yl)pyrrolidine.

1-(1-(Naphthalen-1-yl)-3-phenylprop-2-yn-1-yl)pyrrolidine **(4c)**
White solid, mp = 117–118 ˚C, ^{1}H NMR (400 MHz, $CDCl_3$, ppm): δ 8.33 (d, 8.15 Hz, 1H), 7.83 (d, 1H), 7.77 (d, 1H), 7.72 (d, 1H), 7.57–7.35 (m, 5H), 7.23 (br.s, 3H), 5.49 (s, 1H), 2.71 (s, 2H), 2.61 (s, 2H), 1.68 (s, 4H); ^{13}C NMR (100 MHz, $CDCl_3$, ppm): 134.3, 133.8, 131.8, 130.5, 128.5, 128.4, 128.3, 128.1, 126.0, 126.0, 125.7, 125.0, 124.5, 123.7, 87.4, 86.8, 56.2, 50.2, 24.2; IR (neat) ν_{max}: 3053, 2971, 2878, 2797, 1596, 1508, 1488, 1443, 1397, 1343, 1259, 1128, 1102, 1071, 1043, 955, 914, 851, 802, 779, 757, 692, 638, 563, 513 cm^{-1}.

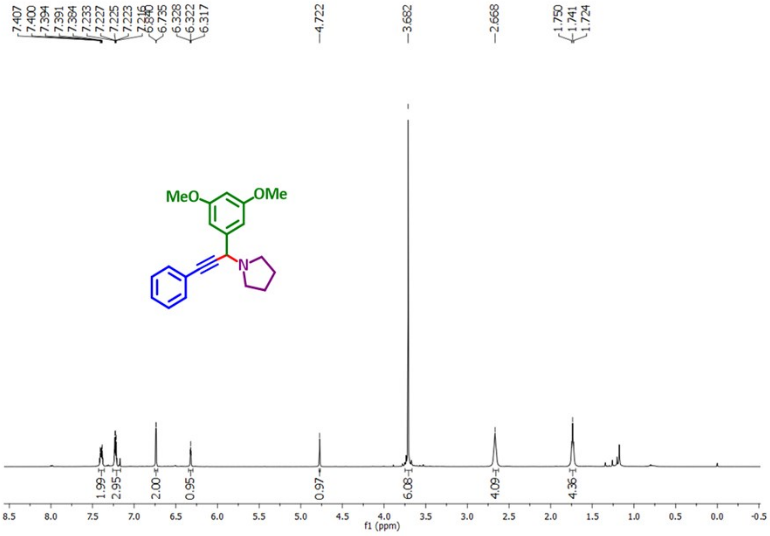

Figure S5 ^{1}H NMR of 1-(1-(3,5-Dimethoxyphenyl)-3-phenylprop-2-yn-1-yl)pyrrolidine.

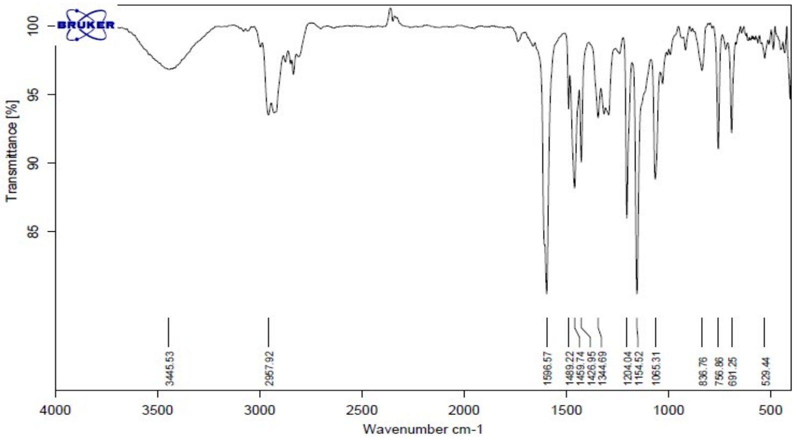

Figure S6 IR spectrum of 1-(1-(3,5-Dimethoxyphenyl)-3-phenylprop-2-yn-1-yl)pyrrolidine.

1-(1-(3,5-Dimethoxyphenyl)-3-phenylprop-2-yn-1-yl)pyrrolidine **(4d)**
Yellowish oil, ^{1}H NMR (400 MHz, $CDCl_3$, ppm): δ 7.40–7.38 (m, 2H), 7.23–7.21 (m, 3H), 7.73 (d, 2H), 6.32 (t, 1H), 4.72 (s, 1H), 3.68 (s, 6H), 2.61–2.77 (m, 4H), 1.74 (t, 5.78 Hz, 4H); ^{13}C NMR (100 MHz, $CDCl_3$, ppm) 160.7, 141.5, 131.8, 128.3, 128.2, 123.1, 106.4, 99.8, 87.0, 86.3, 59.3, 55.4, 50.4, 23.6, IR (neat) ν_{max}: 3080, 2997, 2958, 2930, 2835, 1597, 1489, 1460, 1427, 1345, 1293, 1204, 1155, 1065, 1029, 917, 757, 691 cm^{-1} (Figure S5 and Figure S6).

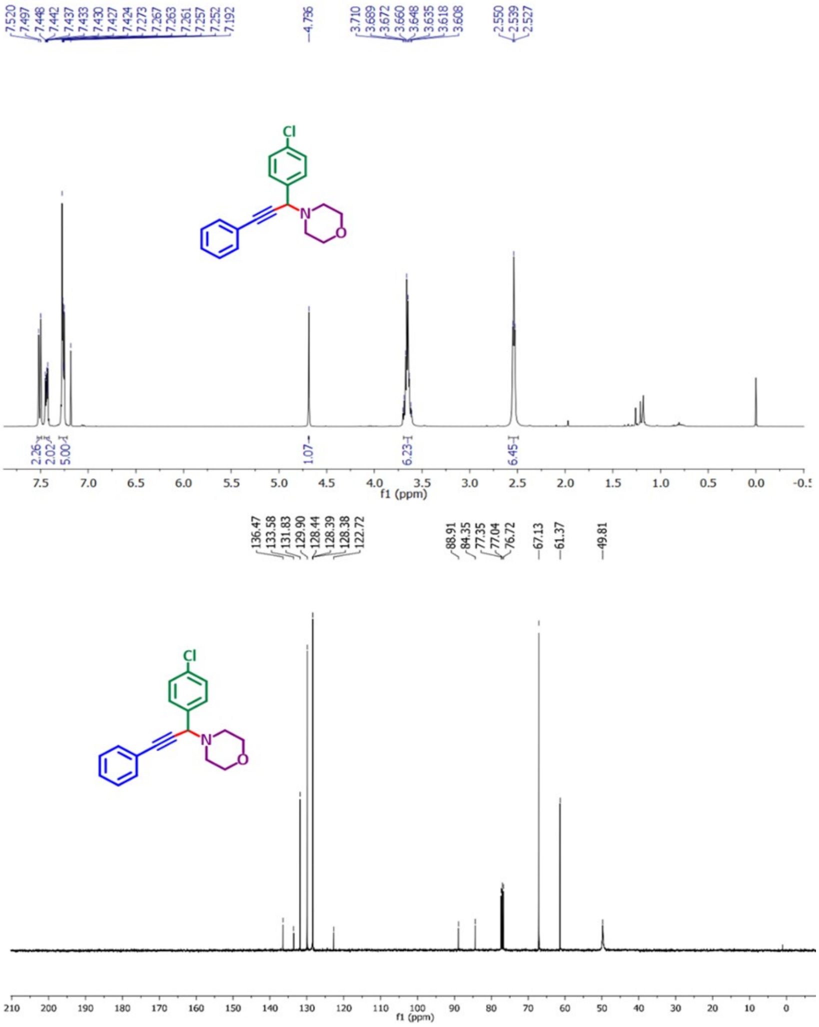

Figure S7 ^{1}H NMR and ^{13}C NMR of 4-(1-(4-Chlorophenyl)-3-phenylprop-2-yn-1-yl)morpholine.

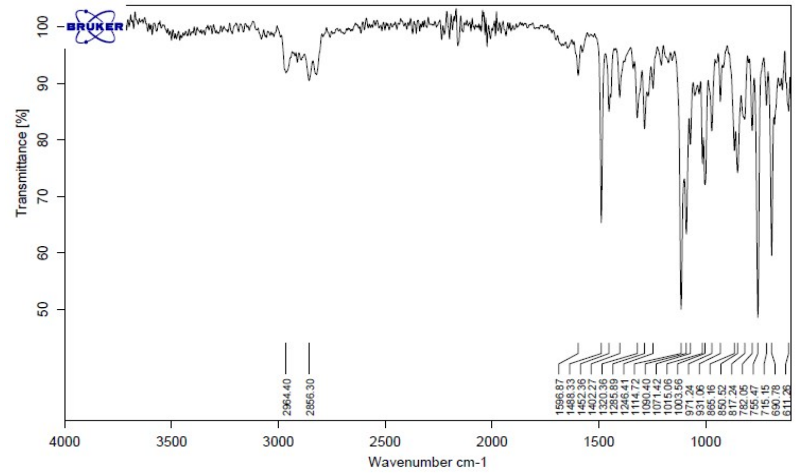

Figure S8 IR spectrum of 4-(1-(4-Chlorophenyl)-3-phenylprop-2-yn-1-yl)morpholine.

4-(1-(4-Chlorophenyl)-3-phenylprop-2-yn-1-yl) morpholine **(4f)**

Yellowish liquid, ^{1}H NMR (400 MHz, $CDCl_3$, ppm): δ 7.52 (d, 2H), 7.44–7.43 (m, 2H), 7.27–7.19 (m, 5H), 4.73 (s, 1H), 3.71–3.61 (m, 4H), 2.55–2.52 (t, 4H); ^{13}C NMR (100 MHz, $CDCl_3$, ppm) 136.5, 133.6, 131.9, 129.9, 128.44, 128.39, 128.38, 122.7, 88.9, 84.3, 67.1, 61.4, 49.8, IR (neat) ν_{max}: 3080, 2964, 2856, 2822, 1597, 1488, 1452, 1402, 1320, 1286, 1115, 1096, 1004, 971, 851, 782, 755, 691 cm^{-1} (Figure S7 and Figure S8).

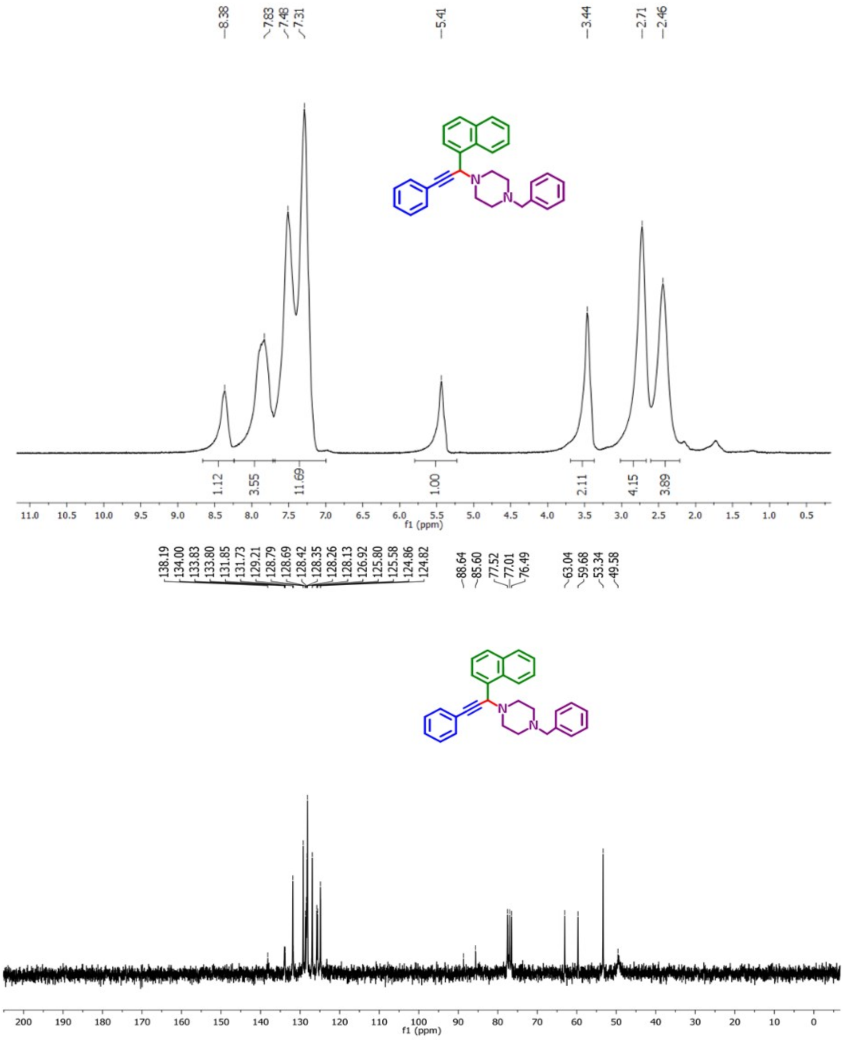

Figure S9 ^{1}H NMR and ^{13}C NMR of 1‐Benzyl‐4‐(1‐(naphthalen‐1‐yl)‐3‐phenylprop‐2‐yn‐1‐yl) piperazine.

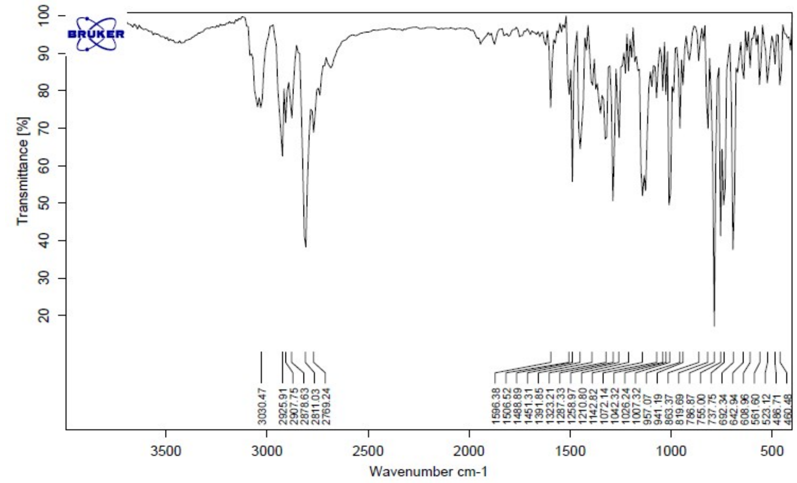

Figure S10 IR spectrum of 1‐Benzyl‐4‐(1‐(naphthalen‐1‐yl)‐3‐phenylprop‐2‐yn‐1‐yl) piperazine.

1‐Benzyl‐4‐(1‐(naphthalen‐1‐yl)‐3‐phenylprop‐2‐yn‐1‐yl) piperazine **(4l)**

White solid, mp = 154–156 ˚C, ^{1}H NMR (400 MHz, $CDCl_3$, ppm): δ = 7.31–8.38 (m, 11H), 5.41 (s, 1H), 3.44 (br, 2H), 2.71 (br, 4H), 2.46 (br, 4H); ^{13}C NMR (100 MHz, $CDCl_3$): 138.19, 134.00, 133.83, 133.80, 131.85, 131.73, 129.21, 128.79, 128.69, 128.42, 128.35, 128.26, 128.13, 126.92, 125.80, 125.58, 124.86, 124.82, 88.64, 85.60, 63.04, 59.68, 53.34, 49.58,; IR (neat) ν_{max}: 3030, 2925, 2907, 2878, 2811, 2769, 1596, 1506, 1488, 1451, 1391, 1323, 1287, 1258, 1210, 1142, 1026, 1007, 957, 819, 786, 755, 692, 523 cm^{-1} (Figure S9 and Figure S10).

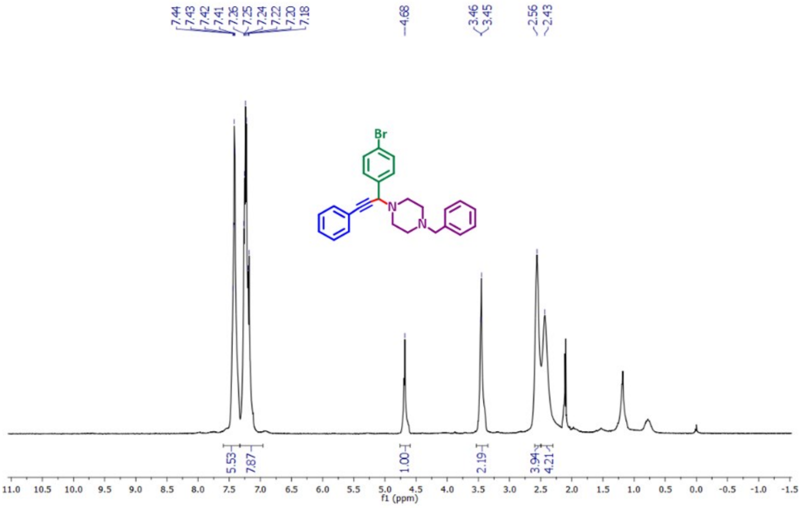

Figure S11 ^{1}H NMR of 1-benzyl-4-(1-(4-bromophenyl)-3-phenylprop-2-yn-1-yl)piperazine.

1-benzyl-4-(1-(4-bromophenyl)-3-phenylprop-2-yn-1-yl)piperazine **(4k)**
White solid, mp = 154–156 ˚C, ^{1}H NMR (250 MHz, $CDCl_3$): δ (ppm) = 7.18–7.44 (m, 14H), 4.68 (s, 1H), 3.45 (br, 2H), 2.56 (br, 4H), 2.43 (br, 4H) (Figure S11).

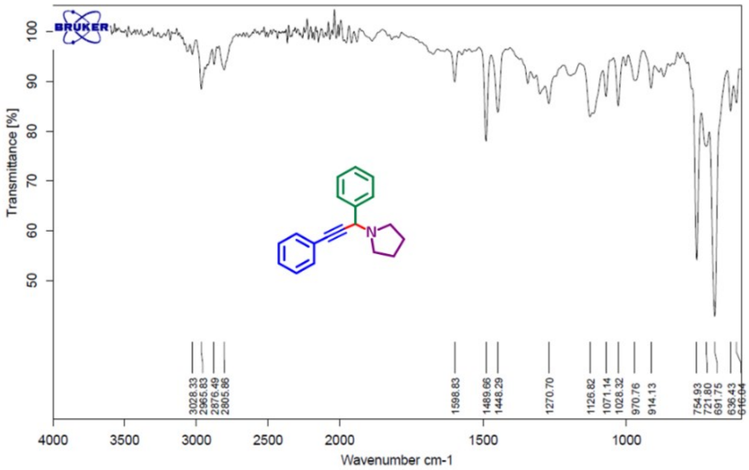

Figure S12 IR spectrum of 1-(1,3-diphenylprop-2-yn-1-yl)pyrrolidine.

1-(1,3-diphenylprop-2-yn-1-yl)pyrrolidine **(4a)**
Yellowish oil, IR (neat) ν_{max}: 3028, 2876, 2805, 1598, 1489, 1448, 1270, 1126, 1071, 1028, 970, 914, 754, 721, 691, 636, 616 cm^{-1} (Figure S12).

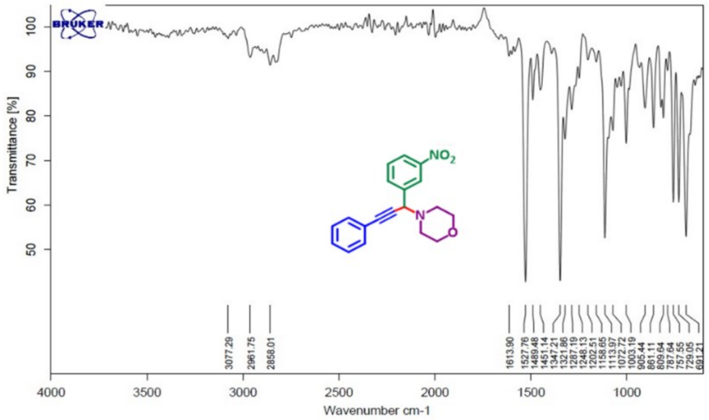

Figure S13 IR spectrum of 1-(1-(3-nitrophenyl)-3-phenylprop-2-yn-1-yl)morpholine.

*1-(1-(3-nitrophenyl)-3-phenylprop-2-yn-1-yl)*morpholine **(4g)**
Yellowish oil, IR (neat) ν_{max}: 3077, 2961, 2858, 1613, 1527, 1489, 1451, 1347, 1321, 1287, 1248, 1202, 1158, 1113, 1072, 1003, 905, 861, 809, 787, 757, 729, 691 cm^{-1} (Figure S13).

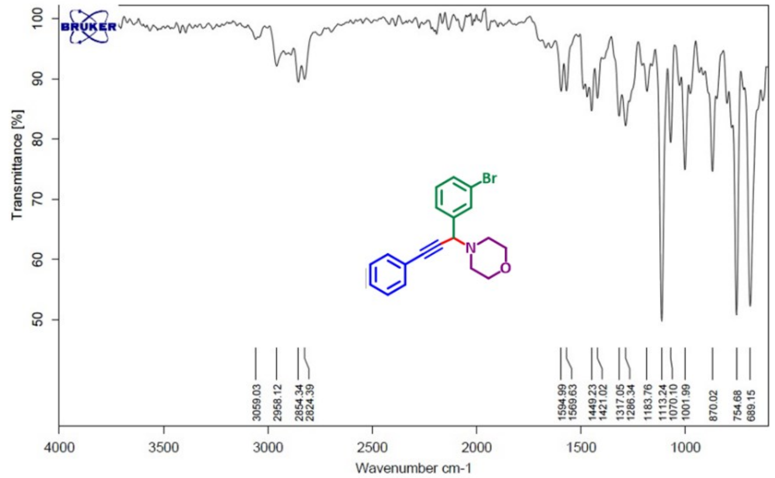

Figure S14 IR spectrum of 4-(1-(4-Bromophenyl)-3-phenylprop-2-yn-1-yl)morpholine.

4-(1-(4-Bromophenyl)-3-phenylprop-2-yn-1-yl)morpholine **(4h)**
Yellowish oil, IR (neat) ν_{max}: 1594, 1569, 1449, 1421, 1317, 1286, 1183, 1113, 1070, 1001, 870, 754, 689 cm^{-1} (Figure S14).

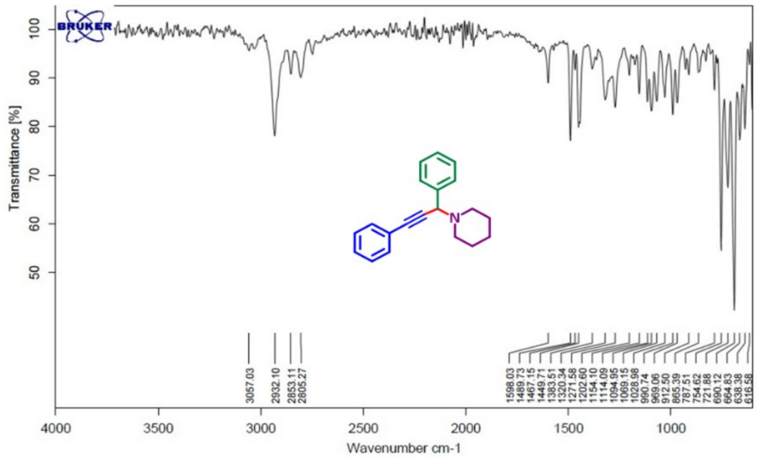

Figure S15 IR spectrum of 1-(1,3-diphenylprop-2-yn-1-yl)Piperidine.

1-(1,3-diphenylprop-2-yn-1-yl) Piperidine **(4i)**
Yellowish oil, IR (neat) ν_{max}: 1598, 1489, 1467, 1449, 1383, 1320, 1271, 1202, 1154, 1114, 1094, 1069, 1028, 990, 969, 912, 865, 787, 754, 721, 690, 664, 638, 616 cm^{-1} (Figure S15).

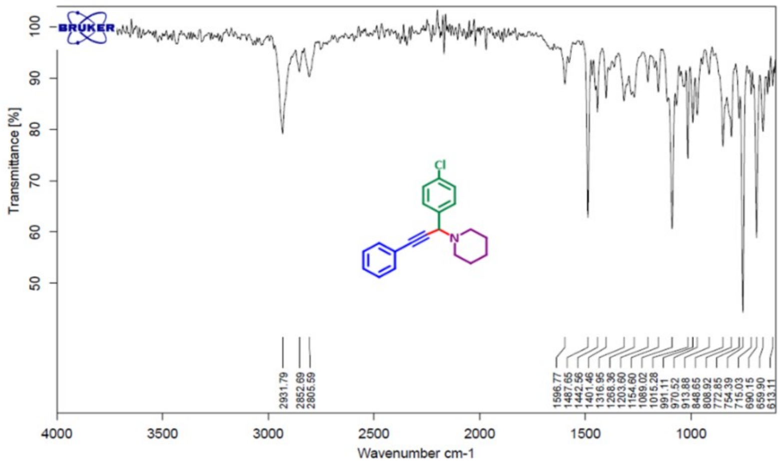

Figure S16 IR spectrum of 4-(1-(4-Chlorophenyl)-3-phenylprop-2-yn-1-yl)Piperidine.

4-(1-(4-Chlorophenyl)-3-phenylprop-2-yn-1-yl)Piperidine **(4j)**
Yellowish oil, IR (neat) ν_{max}: 1596, 1487, 1442, 1401, 1316, 1268, 1203, 1154, 1089, 1015, 991, 970, 913, 848, 808, 772, 754, 715, 690, 659, 613 cm^{-1} (Figure S16).
